# Hematopoietic and T-lymphopoiesis-stimulating effects of a fluorinated pyrazolopiperidine β-cyclodextrin complex in cyclophosphamide-induced hematopoietic suppression

**DOI:** 10.3389/fphar.2026.1852779

**Published:** 2026-08-03

**Authors:** Assel Ten, Layilya Baktybayeva, Zhanargul Koshetova, Raushan Koizhaiganova, Guldana Daulet, Tolganay Zharkynbek, Valentina Yu

**Affiliations:** 1 Laboratory of Synthetic and Natural Medicinal Compounds Chemistry, A. B. Bekturov Institute of Chemical Sciences, Almaty, Kazakhstan; 2 Department of Biophysics, Biomedicine and Neuroscience, Al Farabi Kazakh National University, Almaty, Kazakhstan; 3 Department of Chemistry, Kazakh National Women’s Teacher Training University, Almaty, Kazakhstan; 4 Department of Anatomy, Physiology, and Sports Medicine, Faculty of Olympic Sport, Kazakh Academy of Sport and Tourism, Almaty, Kazakhstan; 5 Department of Analytical, Colloid Chemistry and Technology of Rare Elements, Farabi University, Almaty, Kazakhstan

**Keywords:** cyclophosphamide-induced T-lymphopoiesis suppression, hematopoietic recovery, immunomodulatory activity, pyrazolopiperidine β-cyclodextrin complex (BIV), T-lymphopoiesis stimulating activity

## Abstract

**Background:**

Cyclophosphamide is widely used in oncology, autoimmune disorders, and transplantation-related conditioning or post-transplant immunosuppression. However, its therapeutic value is limited by hematopoietic and lymphoid toxicity, including leukopenia, neutropenia, anemia, thrombocytopenia, bone marrow suppression, thymic injury, and prolonged impairment of T-cell recovery. These effects may occur after pulsed intravenous administration, high-dose regimens used in transplantation or severe refractory disease, and continuous low-dose/metronomic exposure. Therefore, there is a medical need for pharmacological agents capable of compensating cyclophosphamide-induced hematopoietic and lymphopoietic suppression without causing uncontrolled stimulation of early progenitor cells.

**Aim:**

To evaluate the hematopoietic and T-lymphopoiesis-stimulating activity of BIV under cyclophosphamide-induced hematopoietic and T-lymphopoietic suppression.

**Methods:**

Cyclophosphamide-induced hematopoietic- and T-lymphopoiesis-suppressive models were established on laboratory albinos rats and C57BL6/J mice. Peripheral blood hematological indices in rats were measured using hematology analyzers. Hematopoietic stem cells, thymocyte subsets, Th_act_, T_reg_, T_mem_, CTL cell populations, and erythroid cells from the bone marrow, thymus, and spleen were analyzed by flow cytometry. Statistical analysis was performed using one-way ANOVA (p < 0.05).

**Results:**

In rats with CPh-induced hematopoietic suppression, BIV supported partial recovery of erythrocyte and hemoglobin parameters and improved leukopoietic indices under CPh-induced hematopoietic suppression. The β-cyclodextrin complex of 5-benzyl-7-(o-fluorobenzylidene)-2,3-bis(o-fluorophenyl)-3,3a,4,5,6, 7-hexahydro-2H-pyrazolo[4,3-c]pyridine (BIV) demonstrated a consistent moderate activity in restoring the overall level of CD3ε+CD19− T lymphocytes in the bone marrow and thymus. In the bone marrow, BIV uniformly increased the reduced levels of CD3ε+CD19− T lymphocytes and CD3ε+CD44^+^ T_mem_ memory T cells to physiological normal values. In the spleen, BIV restored the decreased levels of CD25+FoxP3+ T_reg_ regulatory lymphocytes and CD28^+^CD44^+^ T_mem_ memory T cells to physiological normal levels, while reducing the elevated levels of CD3ε+CD8a+ CTL cytotoxic T lymphocytes and CD4^+^CD25^+^ Th_act_ activated helper T cells to values corresponding to the physiological norm.

**Conclusion:**

BIV demonstrated notable hematopoietic- and T-lymphopoiesis-modulating activity. The compound partially restored erythropoiesis as well as central and peripheral T-lymphopoiesis, while providing balanced regulation of cytotoxic, regulatory, and memory T-cell subsets in cyclophosphamide-induced T-lymphopoiesis-suppressive models. Several evaluated T-lymphocyte subpopulations approached or returned to physiological control levels under the experimental conditions, although full translational validation requires further pharmacological and toxicological investigation.

## 1 Introduction

Alkylating cytostatics remain integral to conditioning regimens in oncology and allogeneic hematopoietic transplantation (Meade and Bolaños-Meade, 2024). However, the clinical use of cytostatic agents continues to expand each year, and these drugs are increasingly incorporated into treatment protocols in rheumatology, allergology, dermatology, cardiovascular medicine, dentistry, traumatology, transplantation medicine, and cosmetic practice (Kato et al., 2020; Zhu et al., 2020; Haselwanter et al., 2023; Teles et al., 2017; Robinson et al., 2016; Sato-Okabayashi et al., 2020). Cyclophosphamide (CPh) induces profound depression of hematopoietic and lymphoid progenitors with long-lasting cytopenia (Singh et al., 2021). Cyclophosphamide-related hematological toxicity depends on the exposure schedule, cumulative dose, and clinical indication. Pulsed intravenous regimens are commonly used in severe autoimmune and inflammatory diseases to achieve immunosuppression while limiting cumulative exposure; nevertheless, transient leukopenia, neutropenia, lymphopenia, and infection risk remain clinically important. High-dose cyclophosphamide regimens, including transplantation-related protocols and severe refractory immune-mediated conditions, are associated with deeper immune depletion and delayed hematopoietic recovery, even when they are used for selective immunomodulatory purposes. Continuous low-dose or metronomic cyclophosphamide produces a different biological profile, with antiangiogenic and immunomodulatory effects, including suppression of regulatory T-cell compartments, but prolonged exposure may still disturb immune homeostasis and requires hematological monitoring. These regimen-dependent toxicities support the need for adjunctive agents that can compensate cytostatic injury by promoting balanced hematopoietic and lymphopoietic recovery. Cytostatic-induced myelo- and lymphocytodepressive conditions can lead to acute infectious processes, tissue necrosis, and exacerbation of chronic diseases (Guevara et al., 2023; Abdoon et al., 2023; Makheja et al., 2013; Huyan et al., 2011; Angley et al., 1995). Cyclophosphamide (CPh) causes a significant reduction in T-lymphocyte levels. According to the study by Grinko et al. (2020), complete restoration of T-lymphocyte subpopulations was observed only by the 60th day after the initial administration of cyclophosphamide. Prolonged cyclophosphamide-induced T-lymphopoiesis suppression resulted in excessive phenotypic conversion of Tnaive cells into CTLs, as well as accumulation of Th1 cells and autoreactive CTL clones. Numerous studies have emphasized the importance of timely restoration of the mitotic activity of the T-lymphoid pool in the bone marrow, accompanied by adequate repopulation of the thymus and reduction of the risk associated with the accumulation of autoreactive CTLs. However, the range of currently available myelopoiesis- and lymphopoiesis-stimulating agents remains limited, and many of these drugs are associated with pronounced adverse effects. synthetic and naturally derived nucleic acid–based agents, including sodium nucleinate, pentoxyl, poludan, inosine pranobex, methyluracil, and inosine, may stimulate the growth and proliferation of prokaryotic, benign, and malignant cells (Dressel, 1977; Klinman et al., 1996; Qu et al., 2024). The immunostimulatory activity of CpG oligodeoxynucleotides depends on their structure and delivery system (Hanagata, 2012). First-generation thymic agents are no longer used in therapeutic practice. Second-generation thymic preparations, including Thymosin, Thymopoietin, Timalin, Thymostimulin, Vilozen, Thymomodulin, Timurovich, and Thymulin, are generally administered only under inpatient conditions and may induce severe adverse effects, including septic shock, destruction of host tissues, and autoimmune disorders (Liu et al., 2020). Myelopeptide-based agents, including Filgrastim, Molgramostim, Lenograstim, Pegfilgrastim, Lipegfilgrastim, and Empegfilgrastim, exhibit a broad spectrum of biological activity. However, these agents may induce uncontrolled pyrogenic reactions, severe bone pain, and other systemic adverse effects. Consequently, a dose adjustment and administration must be performed strictly under medical supervision in clinical settings (Lee et al., 2020; Di Carlo et al., 2022; Thwaites et al., 2021; Zhao et al., 2021; Papalois et al., 2019; Lundqvist et al., 2013). Cytokine-based preparations, including Leukinferon, Superlymph, Betaleukin, and Roncoleukin, are also administered exclusively under inpatient conditions and require highly precise dosing at extremely low concentrations (10^−6^–10^–12^). Cytokines may induce severe adverse reactions, including septic shock, reduced contractility of the myocardium and vascular smooth muscle cells, increased endothelial permeability, impaired organ microcirculation, hypotension, and hypoglycemia (Finkel et al., 1992; Aoki and Xing, 2004; Azuma, 1992; Liu et al., 2010). Plant-derived adaptogens (Immunal, Echinacea liquidum, Echinacea compositum S, Echinacea VILAR, the root of ginseng, Siberian ginseng) including polysaccharide-rich extracts from medicinal plants display myeloprotective and anti-inflammatory activity (Grafeneder et al., 2022).

Currently, there is an interest in synthetic compounds with a heterocyclic core. Heterocyclic nuclei form the basis for the construction of numerous homological series containing hydrocarbon residues in the form of side chains, as well as various functional groups. The most important are pyrrol, pyrazole, imidazole, pyridine, pyrimidine, piperidine, furan, thiophene, indole, quinoline, purine, benzimidazole, etc (Luo et al., 2024; Baktybayeva et al., 2023; Eryanti et al., 2015; Purygin et al., 2004). Of the registered medicines, 6% are compounds with a pyridine ring and its derivatives. These are anti-tuberculosis, antihypertensive, antihistamines, cardiovascular drugs, antidotes of toxic organophosphorus compounds, neuroleptics and other drugs. The first prerequisite for the synthesis of a new compound was the manifestation of leukopoiesis-stimulating activity with the drug Mexidol (2-ethyl-6-methyl-3-hydroxypyridine succinate). Mexidol is a nootropic, cerebroprotective, stress-protective, antihypoxic, antioxidant, anticonvulsant, anxiolytic and membrane-protective drug. When used in postoperative practice, an increase in the proportion of examined patients with vegetative equilibrium was revealed (from 62.86% to 88.57%; *p* < 0.05), normalization of vegetative reactivity (from 8.57% to 34.29%; *p* < 0.05), blood leukogram analysis showed effective positive dynamics in restoring granulocyte and B-lymphocyte indices, an increase in IgM indices (from 1.89 g/L to 2.12 g/L; *p* < 0.05) with a simultaneous tendency to increase IgA and IgG (Kabdualiev et al., 2007; Tolkacheva et al., 2021). The second prerequisite for the synthesis of a new compound is the selection of an appropriate heterocyclic scaffold, as heterocyclic frameworks, particularly nitrogen-containing systems, dominate the current portfolio of approved small-molecule drugs, including many agents introduced in recent years (Luo et al., 2024; Baktybayeva et al., 2023; Yu et al., 2018). Dienone derivatives of piperidine obtainable through aldol-crotonic condensation yield α,α1-divinyl ketones suitable for construction of polycyclic structures with a piperidine-pyrazoline pharmacophore. Structure-activity data suggested replacement of methoxy substituents with fluorine atoms within the pyrazolopiperidine core would produce a substrate with strong leukopoiesis-stimulating capacity.

In a recent study, we synthesized the β-cyclodextrin inclusion complex of 5-benzyl-7-(o-fluorobenzylidene)-2,3-bis(o-fluorophenyl)-3,3a,4,5,6,7-hexahydro-2H-pyrazolo[4,3-c]pyridine, hereafter referred to as BIV, and evaluated its hematopoietic effects in a rat model of CPh-induced hematopoietic suppression and in a mice model of CPh-induced B-lymphopoietic suppression (Koshetova et al., 2025). BIV accelerated recovery of erythroid, myeloid and lymphoid cells lineages, with marked stimulation of B-lymphocyte maturation and a favorable toxicity profile (Koshetova et al., 2025). The structure is shown in Supplementary Material Figure S1.

Methyluracil (MU) (as control medicine) (2,4-dioxo-6-methylpyrimidine) remains a widely used and inexpensive hematopoietic stimulant in the therapeutic practice in our country (Gimadieva et al., 2013; Ministry of Healthcare of the Republic of Kazakhstan, 2020). Methyluracil enhances the growth and reproduction of cells, improving the course of regeneration in the damaged tissues, accelerates the healing of wounds, ulcers, burns, and increases the body’s resistance to infections. A characteristic feature of the preparation is its stimulating effect on hematopoiesis (an increased formation of leukocytes and red blood cells in the bone marrow). Besides, methyluracil has an anti-inflammatory effect, increases the body’s resistance to the blood loss and oxygen deficiency, and normalizes the production of gastric juice and its acidity.

Based on these data, BIV represents a promising candidate for targeted restoration of the hematopoiesis and T-lymphopoiesis in cyclophosphamide-induced hematopoiesis- and T-lymphopoiesis-suppressive conditions. The present work aimed to evaluate whether BIV effectively restores hematopoiesis in a rat model of CPh-induced hematopoietic suppression and T-lymphopoiesis in a mouse model of CPh-induced T-lymphopoiesis suppression.

## 2 Materials and methods

### 2.1 General considerations and preparation of compounds

In the biological experiments, the β-cyclodextrin inclusion complex of 5-benzyl-7-(o-fluorobenzylidene)-2,3-bis(o-fluorophenyl)-3,3a,4,5,6,7-hexahydro-2H-pyrazolo[4,3-c]pyridine (PPβCD), previously described and characterized by Koshetova et al. (2025), was used under the internal experimental code “BIV”. Accordingly, the designation BIV used in figures and biological assays throughout this work refers to the PPβCD complex. The synthesis of the key intermediates and the preparation of the inclusion complex PPβCD (BIV, including reaction monitoring, purification, and structural confirmation by TLC, elemental analysis, melting point determination, IR, and NMR spectroscopy) were performed as described in our previous work (Koshetova et al., 2025) and are not repeated here.

### 2.2 Biological research methods

#### 2.2.1 Design of the experiment on hemostimulating activity (rats)

The hemostimulating activity of BIV was evaluated using adult laboratory white rats. The experimental design for investigating hemostimulatory activity using a cyclophosphamide-induced hemosuppression model is widely employed in many laboratories worldwide (Bondarchuk, 2016). Animals were housed in a pathogen-free barrier facility under controlled conditions (21 °C–23 °C) with free access to standard laboratory chow and water. Lighting conditions were maintained under a natural light–dark cycle without disruption of the animals’ circadian rhythms. The physiological condition of the animals, including body weight, coat condition, and stool characteristics, was continuously monitored throughout the study. The animals were housed in a sanitary protection area with *ad libitum* access to purified water supplied through automatic drinking systems and standardized pelleted feed. The study included nulliparous female rats weighing 180–220 g and aged 8–9 weeks. The animals belonged to the outbred SD (Sprague Dawley) albino rat strain and were obtained from the “Pushchino” Laboratory Animal Breeding Facility (M.M. Shemyakin and Yu.A. Ovchinnikov Institute of Bioorganic Chemistry, Russia). The animal population is maintained at the Biological Clinic of the Department of Biology and Biotechnology, Farabi University. Animals were randomly divided into four experimental groups: untreated/intact (UT), placebo (PL), BIV, and methyluracil (MU). Each group consisted of six animals. Hematopoietic suppression was induced by administration of the cytostatic agent cyclophosphamide (Baxter Oncology GmbH, Germany). On experimental days 1, 3, and 5, animals in the PL, MU, and BIV groups received intramuscular injections of a 3% cyclophosphamide solution at a dose of 30 mg/kg, prepared in isotonic sodium chloride solution. The average injection volume ranged from 0.2 to 0.24 mL. Animals in the UT group did not receive any injections. From days 6–8 of the experiment, all administrations were performed at 09:00 a.m. Rats in the BIV group received BIV dissolved in saline at a dose of 10 mg/kg, administered intramuscularly in a volume of 0.2–0.25 mL (1% solution). The therapeutic dose of BIV was established at 10 mg/kg, based on the relationship ED_50_(BIV) = 1/100 × LD_50_(BIV). The acute toxicity study demonstrated that LD_50_(BIV) was 754.21 ± 12.54 mg/kg, as previously reported by Koshetova et al. (2025) for the fluorinated pyrazolopiperidine inclusion complex. The 1/100 fraction of the LD50 value was selected to provide a broader therapeutic window and to ensure a favorable safety margin for BIV administration. Pharmacokinetic studies of BIV are currently being conducted in 2026. The pharmacokinetic evaluation was intentionally initiated after completion of the preliminary safety and efficacy assessments, as confirmation of the compound’s biological activity and toxicological safety was considered essential before proceeding to detailed ADME (absorption, distribution, metabolism, and excretion) investigations. The pharmacokinetic data are expected to be published in 2027.

Animals in the MU group received 6-methyluracil under identical conditions and at the same dose and volume. The PL group received an equivalent volume of saline only. The UT group remained untreated throughout the study. On day 15 of the experiment, corresponding to 7 days after the last administration, blood samples were collected at 09:00 a.m. from the orbital sinus under general anesthesia using ketamine (91.0 mg/kg) and xylazine (9.1 mg/kg). Blood was collected into EDTA (K2) hematology tubes (2 mL) (Siny Medical EDTA Tube, China) (Baktybayeva et al., 2023). Animals were fasted for 12 h prior to blood sampling.

Hematological analysis was performed using a MicroCC-20 Plus automated animal hematology analyzer (HTI, USA). The following parameters were determined: total white blood cell count (WBC); absolute and relative counts of lymphocytes (LYM), monocytes (MON), neutrophils (NEU), eosinophils (EO), and basophils (BAS); red blood cell count (RBC); hemoglobin concentration (HGB); hematocrit (HCT); mean corpuscular volume (MCV); mean corpuscular hemoglobin (MCH); mean corpuscular hemoglobin concentration (MCHC); red blood cell distribution width (RDW-cv and RDW-sd); platelet count (PLT); thrombocrit (PCT); and mean platelet volume (MPV).

#### 2.2.2 Design of the experiment on T-lymphopoiesis-stimulating activity (mice)

Female C57BL6/J mice, obtained from Charles River Laboratories (USA) and propagated at the Kazakh Scientific Center for Quarantine and Zoonotic Infections named after Aikimbayev, were used in this study. The experimental design for evaluating leukopoiesis-stimulating activity in mice using a cyclophosphamide-induced leukopoiesis suppression model is widely employed in many laboratories worldwide (Huyan et al., 2011; Tian et al., 2021; He et al., 2018; Yang et al., 2019)

Animals were housed in a pathogen-free barrier facility under controlled conditions (21 °C–23 °C) with free access to standard laboratory chow and water. Lighting conditions were maintained under a natural light–dark cycle without disruption of the animals’ circadian rhythms. The physiological condition of the animals, including body weight, coat condition, and stool characteristics, was continuously monitored throughout the study. The animals were housed in a sanitary protection area with *ad libitum* access to purified water supplied through automatic drinking systems and standardized pelleted feed. Nulliparous female mice weighing 20–24 g and aged 8–9 weeks were selected for the experiment.

Mice were randomly allocated into four experimental groups: UT, PL, BIV, and MU, with six animals per group. The experimental design is schematically illustrated in Supplementary Material Figure S2. To induce lymphohematopoietic suppression, cyclophosphamide (Baxter Oncology GmbH, Germany) was dissolved in isotonic saline and administered intramuscularly to mice in the BIV, MU, and PL groups on days 1, 3, and 5 of the experiment at 09:00 a.m. The dose was 100 mg/kg, corresponding to approximately 0.025 mL of an 8% solution. Animals in the UT group did not receive any injections. On days 8, 10, and 12 of the experiment, at 09:00, mice in the BIV group were administered BIV dissolved in saline at a dose of 10 mg/kg (0.025 mL of a 1% solution, intramuscularly). Animals in the MU group received 6-methyluracil under identical conditions and at the same dose and volume. The PL group received an equivalent volume of saline (0.025 mL), while the UT group remained untreated throughout the study. On day 15, following completion of the treatment course, animals were anesthetized with ketamine (87.5 mg/kg) and xylazine (12.5 mg/kg). Hind limb bones and spleen were surgically collected, after which the animals were sacrificed by decapitation for further analysis.

All experimental procedures were conducted in accordance with the *Rules for Conducting Preclinical (Nonclinical) Studies of Biologically Active Substances* and the *Ethical Principles and Recommendations for Scientific Experiments on Animals* (Ministry of Healthcare of the Republic of Kazakhstan, 2020). The study protocol was approved by the Local Ethics Commission of Al-Farabi Kazakh National University (Protocol No. 470, 10 June 2022; Protocol #IRB-A125) and remains valid until 8 October 2025.

### 2.3 Preparation of phosphate-buffered saline (PBS) and column buffer

PBS was used for cell washing and for preparation of the column buffer. PBS was prepared by dissolving NaH_2_PO_4_ and NaCl in purified water and adjusting pH to 7.4 using NaOH. The column buffer was prepared by adding 0.5% FBS (fetal bovine serum) and 0.002 M EDTA to PBS. All solutions were sterile-filtered (0.22 µm) and stored at 4 °C–8 °C until use.

### 2.4 Bone marrow, thymus, and spleen cellularity assessment

Bone marrow, thymus, and spleen cell suspensions were prepared using PBS. Erythrocytes were lysed with a solution containing 0.83% NH_4_Cl, 0.1% KHCO_3_, and 0.003% EDTA (pH 7.2–7.4) for 10 min at room temperature. Cells were then washed, passed through 30 µm pre-separation filters (Miltenyi Biotec, Bergisch Gladbach, Germany), resuspended in PBS, counted, and assessed for viability using trypan blue (Grafeneder et al., 2022).

### 2.5 Cytometric studies of bone marrow, thymus, and spleen

A forward- and side-scatter gating strategy was used to exclude debris. Sample preparation and acquisition were performed using a rapid workflow with strict control of temperature and pH throughout all stages. The following mouse monoclonal antibodies (mAbs) were used for surface staining: Anti-Mouse CD117 (c-Kit) Monoclonal Antibody (2B8(RUO)), APC, BD Pharmingen™ Cat # 553356, 100 μg; Anti-Mouse CD43 Monoclonal Antibody (S7(RUO)), FITC BD Pharmingen™ 100 µg Cat #553270; Anti-Mouse CD19 Monoclonal Antibody (1D3 (RUO)), PerCP-CyTM5.5, BD PharmingenTM 100 µg Cat #551001; Anti-Mouse CD3*ε* Monoclonal Antibody (145-2C11(RUO)), PE, BD Pharmingen™ 100 µg Cat #553063; Anti-Mouse CD4 Monoclonal Antibody (RM4-5), FITC, BD Pharmingen™ 100 µg Cat #553047; Anti-Mouse CD8a Monoclonal Antibody (53–6.7(RUO)), PerCP-Cy™5.5, BD Pharmingen™ 100 µg Cat #553036; Anti-Mouse CD25 Monoclonal Antibody (PC61(RUO)), PerCP-Cy™5.5, BD Pharmingen™ 50 µg Cat #551071; Anti-Mouse FoxP3 Monoclonal Antibody (MF23(RUO)), PE, eBioscience™ 50 µg Cat #560408; Anti-Mouse CD28 Monoclonal Antibody (37.51), PerCP-Cy™5.5, eBioscience™ 50 µg Cat #16–0281–82; Anti-Mouse CD44 Monoclonal Antibody (IM7(RUO)), FITC, BD Pharmingen™ 50 µg Cat #553133; Anti-Mouse TER-119 Monoclonal Antibody (TER-119(RUO), PerCP-Cy™5.5, BD Pharmingen™ 50 µg Cat #560512; Anti-Mouse CD71 Monoclonal Antibody [RI7 217.1.3), APC, BD Pharmingen™ 50 µg Cat # 567258.

The following subpopulations of cells were studied: CD117^+^ - bone marrow stem cells with activated tyrosine kinase activation pathway; CD43^+^CD3ε^+^CD19^−^ - early pre-T lymphocytes; CD3ε^+^CD4^+^ - Th-helper lymphocytes; CD3ε^+^CD8a^+^ - CTL-cytotoxic T-lymphocytes; CD4^+^CD25^+^ - Тh_act_-activated helper lymphocytes; CD4^+^CD25^+^FoxP3^+^ - T-regulatory lymphocytes; CD28^+^CD8a^+^CD44^+^ - Т_mem_-co-stimulated memory cells, Ter119^+^CD71^+^ - erythroid cells.

Briefly, 1 × 10^6^ cells were incubated with the indicated antibodies according to manufacturer protocols and fixed using BD fixation solution (cat. No. 554722) for 20 min at room temperature in the dark. Cells were washed, resuspended in flow solution, and analyzed immediately on a FACSCalibur flow cytometer (BD Biosciences, San Jose, CA, USA) using CellQuest Pro software (BD Biosciences, San Jose, CA, USA). Unstained cells, single-stained controls, and fluorescence minus-one controls were used for instrument setup.

### 2.6 Statistical data processing

Data were processed using Microsoft Excel and Statistica 6.0. Results are presented as mean (M) ± standard deviation (SD) for n = 6 mice per group. Statistical differences among groups were evaluated using one-way analysis of variance (ANOVA), with significance accepted at p < 0.05 and F > Fcrit. One-way ANOVA was selected because the experimental animals were of the same sex, belonged to the same strain, and had highly comparable body weights. Animal husbandry conditions, including diet and care, complied with SPF (Specific Pathogen Free) standards and were maintained according to the experimental protocol. Experimental repetitions (performed at least twice) were conducted during the same season of the year under identical circadian conditions, while ambient temperature and humidity were maintained at constant levels throughout the study. For predefined pairwise comparisons among experimental groups, exact p-values and F statistics were reported in the corresponding Results sections, tables, and figure legends. Because the study was exploratory and based on a fixed preclinical group size of six animals per group, the findings were interpreted cautiously, and statistical significance was not treated as standalone evidence of therapeutic efficacy.

### 2.7 Graphical methods of data visualization

Chemical structures were prepared using ChemDraw (PerkinElmer Informatics). Graphs were generated using GraphPad Prism 9.2.0. Experimental design figures were created using BioRender.

## 3 Results

### 3.1 Hematopoiesis-stimulating activity of BIV

In the CPh‐induced hematopoiesis suppression model, BIV increased bone marrow nucleated cell counts. The mean nucleated cell number in the BIV group reached (72.9 ± 5.18) × 10^6^ cells. This value exceeded the UT group level of (25.1 ± 1.01) × 10^6^ by 2.94-fold. The value also exceeded the PL group level of (55.40 ± 2.81) × 10^6^ by 1.33-fold (Table 1). The effect matched the MU reference group.

**TABLE 1:** Bone marrow cellularity after CPh-induced hematopoietic suppression in mice.

Groups	The average number of the nucleated cells (NC), ×10^6^ cells	P
UT	25.1 ± 7.4	P_UT-*BIV*_ = 0.00006, F > F crit, 94.66 > 5.98
MU	57.4 ± 4.2	P_MU-*BIV*_ = 0.04, F > F crit, 6.05 > 5.98
BIV	72.9 ± 5.1	​
PL	55.4 ± 2.8	P_PL-_ _*BIV*_ = 0.01, F > F crit, 10.36 > 5.98

* *Bone marrow nucleated cell counts for untreated (UT), methyluracil (MU), BIV, and placebo (PL) groups. Data are presented as mean ± SD (n = 6 animals/group). Units: ×106 cells. P values and F statistics are listed*.

Anemia occurs in over 45% of oncology patients before chemotherapy initiation (Sharma et al., 2022). Post-chemotherapy anemia occurs in all patients. CPh-induced hematopoietic suppression serves as a standard model for hematopoietic activity assessment (Abireh et al., 2020). Following CPh exposure, BIV restored hemoglobin concentration to (125.5 ± 3.0) g/L. This level approached the MU group value of (139.5 ± 12.1) g/L and the UT group value of (158.5 ± 16.5) g/L (Table 2). MU remains widely used for correction of CPh-induced hematopoietic depression in oncotherapy (Bondarchuk, 2016). BIV showed higher hematologic recovery across several indices.

**TABLE 2:** Peripheral blood hemogram parameters after CPh-induced hematopoietic suppression in rats.

No	Groups	WBC, ·10^9^/L	LYM, ·10^9^/L	NEU, ·10^9^/L	MON, ·10^9^/L	EO, ·10^9^/L	BAS, ·10^9^/L	LYM, %	NEU, %	MON, %	EO, %	BAS, %	RBC, ·10^12^/L	HGB, g/L	HCT, %	MCV, fL	MCH, pg	MCHC, g/L	RDWsd, fL	RDWcv	PLT, ·10^9^/L
1	BIV	8.1 ± 1.5	5.4 ± 0.1	2.6 ± 0.0	0.1 ± 0.0	0.0 ± 0.0	0.0 ± 0.0	66.2 ± 3.2	32.2 ± 5.3	1.6 ± 0.2	0.0 ± 0.0	0.0 ± 0.0	6.0 ± 0.3	125.5 ± 3.0	31.9 ± 1.2	52.8 ± 0.8	20.8 ± 0.6	394.0 ± 5.3	24.7 ± 0.4	12.85 ± 0.0	850.0 ± 22.0
2	MU	7.2 ± 1.2	4.5 ± 0.1	2.1 ± 0.6	0.3 ± 0.0	0.2 ± 0.0	0.0 ± 0.0	62.8 ± 1.7	29.8 ± 0.6	4.2 ± 0.7	2.8 ± 0.0	0.4 ± 0.0	7.4 ± 1.1	139.5 ± 12.1	30.2 ± 2.3	40.8 ± 1.0	18.7 ± 1.0	459.0 ± 22.5	10.1 ± 0.0	17.0 ± 1.0	340.2 ± 26.1
3	PL	3.8 ± 0.9	1.5 ± 0.1	1.6 ± 0.1	0.47 ± 0.2	0.0 ± 0.0	0.0 ± 0.0	40.6 ± 1.4	44.4 ± 1.6	12.2 ± 0.9	2.0 ± 0.0	0.8 ± 0.0	4.0 ± 1.6	71.0 ± 6.0	11.0 ± 0.31	26.9 ± 1.6	17.4 ± 0.0	647.0 ± 28.8	11.5 ± 0.0	31.2 ± 0.3	381.0 ± 19.6
4	UT	10.7 ± 1.1	6.7 ± 1.5	3.1 ± 0.7	0.3 ± 0.0	0.4 ± 0.0	0.2 ± 0.0	62.4 ± 3.1	29.3 ± 1.0	2.7 ± 0.0	3.5 ± 0.8	3.0 ± 0.0	7.1 ± 1.1	158.5 ± 16.5	36.9 ± 3.2	43.5 ± 2.3	19.4 ± 1.6	446.5 ± 16.5	19.8 ± 4.6	20.9 ± 2.0	561.2 ± 12.2
5	P, F	*	**	***	​	​	​	****	*****	******	​	​	​	*******	********	​	​	​	​	​	​
6	* (P_1-2_ = 0.02 F > F_crit_, 40.5 > 18.51), (P_1-3_ = 0.001 F > F_crit_, 924.5 > 18.51) ** (P_1-2_ = 0.02 F > F_crit_, 40.5 > 18.51), (P_1-3_ = 0.0006, F > F_crit_, 1521 > 18.51) *** (P_1-2_ = н.з.), (P_1-3_ = 0.01 F > F_crit_, 50 > 18.51) **** (P_1-2_ = н.з.), (P_1-3_ = 0.001 F > F_crit_, 578 > 18.51), (P_3-4_ = 0.00003 F > F_crit_, 32768 > 18.51) ***** (P_1-2_ = 0.003 F > F_crit_, 288 > 18.51), (P_1-4_ = 0.0001 F > F_crit_, 7442 > 18.51) ****** (P_1-2_ = 0.002 F > F_crit_, 338 > 18.51), (P_1-3_ = 0.0001 F > F_crit_, 5618 > 18.51) *******(P_1-2_ = 0.001 F > F_crit_, 572 > 18.51), (P_1-3_ = 0.0001 F > F_crit_, 7768 > 18.51) ********(P_1-2_ = н.з.), (P_1-3_ = 0.0003 F > F_crit_, 6264 > 18.51)																				

The data are presented as mean (M) ± standard deviation (SD) (n = 6 rats/group). One-way analysis of variance (ANOVA) has been used to determine statistically significant difference, and values have been considered reliable at p < 0.05 and F > F_cri_.

The erythrocyte count in the BIV group reached (6.0 ± 0.3) × 10^12^/L. This value aligned with MU and UT group levels, each at (7.4 ± 1.1) × 10^12^/L and (7.1 ± 1.1) × 10^12^/L and exceeded the PL group level of (4.0 ± 1.6) × 10^12^/L (Table 2). Mean corpuscular hemoglobin reached (20.8 ± 0.6) pg, which aligned with the UT group value of (19.4 ± 1.6) pg. Mean corpuscular hemoglobin concentration reached (394.0 ± 5.3) g/L, which remained below the UT group value of (446.5 ± 16.5) g/L and the MU group value of (459.0 ± 22.5) g/L (Table 2). The PL group value reached (647.0 ± 28.8) g/L. Mean corpuscular volume reached (52.8 ± 0.8) fL, exceeding the UT group value of (43.5 ± 2.3) fL and the MU group value of (40.8 ± 1.0) fL. RDW-SD reached (24.7 ± 0.4) fL, exceeding the MU group value of (10.1 ± 0.0) fL (Table 2). Peripheral blood smears prepared using Giemsa/Romanowsky-type staining indicated regenerative hematopoiesis with reticulocytes and occasional normoblasts in circulation (Giemsa, 1904). Reticulocyte index ranged from 4.9% to 6.8%.

Leukocyte recovery supports treatment tolerance and infection risk reduction (Tian et al., 2021). Several interventions remain under evaluation for CPh-induced leukopenia (Liu et al., 2017). BIV improved leukogram parameters. Total leukocyte count reached (8.1 ± 1.5) × 10^9^/L. This value exceeded the MU group value of (7.2 ± 1.2) × 10^9^/L and exceeded the placebo group value of (3.8 ± 0.9) × 10^9^/L (Table 2). Neutrophil proportion reached (32.2 ± 5.3)%, which remained below the PL group value of (44.4 ± 1.6)%. Lymphocyte proportion reached (66.2 ± 3.2)%, exceeding the MU, UT, and PL groups. Absolute lymphocyte count reached (5.4 ± 0.1) × 10^9^/L. This value exceeded the PL group value of (1.5 ± 0.1) × 10^9^/L and approached the UT group value of (6.7 ± 1.5) × 10^9^/L (Table 2). Peripheral blood smears indicated myeloid regeneration with circulating immature forms.

Platelet recovery accelerated in the BIV group. Platelet count reached (850.5 ± 22.0) × 10^9^/L. Corresponding values reached (340.2 ± 26.1) × 10^9^/L in the MU group, (561.2 ± 12.2) × 10^9^/L in the UT group, and (381.0 ± 19.6) × 10^9^/L in the PL group (Table 2). Thrombocrit increased in the BIV group.

CPh remains widely used for lymphoma, multiple myeloma, leukemia, ovarian cancer, breast cancer, small cell lung cancer, neuroblastoma, and sarcoma. The agent also serves as a potent immunosuppressant in bone marrow transplantation and several immune-mediated conditions. CPh metabolism includes aldehyde dehydrogenase-mediated inactivation, which contributes to selective cytotoxicity. Differential aldehyde dehydrogenase expression influences therapeutic index and immunosuppressive effects (Steinbrecht et al., 2020). Bone marrow suppression occurs during exposure. T- and B-lymphocytes show high sensitivity (Quan et al., 2022). Mouse models describe CPh use for leukopoiesis-depressive syndromes (Huyan et al., 2011; Nagai, 2023).

The present study assessed BIV effects on hematopoiesis and T-lymphopoiesis in CPh-induced depression. BIV restored nucleated cell levels in bone marrow, thymus, and spleen. Activity matched MU reference values.

### 3.2 T-lymphopoiesis-stimulating activity of BIV under cyclophosphamide-induced T-Lymphopoiesis suppression. Effect of BIV on hematopoietic stem cells and T-Lymphocyte populations in the bone marrow

BIV demonstrated limited stimulatory activity toward hematopoietic stem cells in the bone marrow (Supplementary Material Figure S3). The proportion of CD117^+^ HSCs in the BIV group (4.36 ± 0.10) % did not differ from the PL group (4.57 ± 0.60) %. In contrast, MU increased the CD117^+^ HSC level by 1.25-fold relative to BIV and by 1.38-fold relative to PL (P = 0.004, F > F_crit_, 220.5 > 18.51), although it remained lower than the UT group (7.62% ± 1.61%; P = 0.00007, F > F_crit_, 13944.5 > 18.51).

A similarly modest effect of BIV was observed for CD43^+^CD3ε+T-lymphocytes (Supplementary Material Figure S4). The percentage of these cells in the BIV group reached 15.73% ± 1.54%, which was 2.01-fold lower than in the MU group (35.60 ± 1.34) %(P = 0.0000004, F > F_crit_, 14254.5 > 18.51) but 1.24-fold higher than in the PL group (12.06 ± 1.87) % (P = 0.0001, F > F_crit_, 186.5 > 18. 51).

**FIGURE 1:**
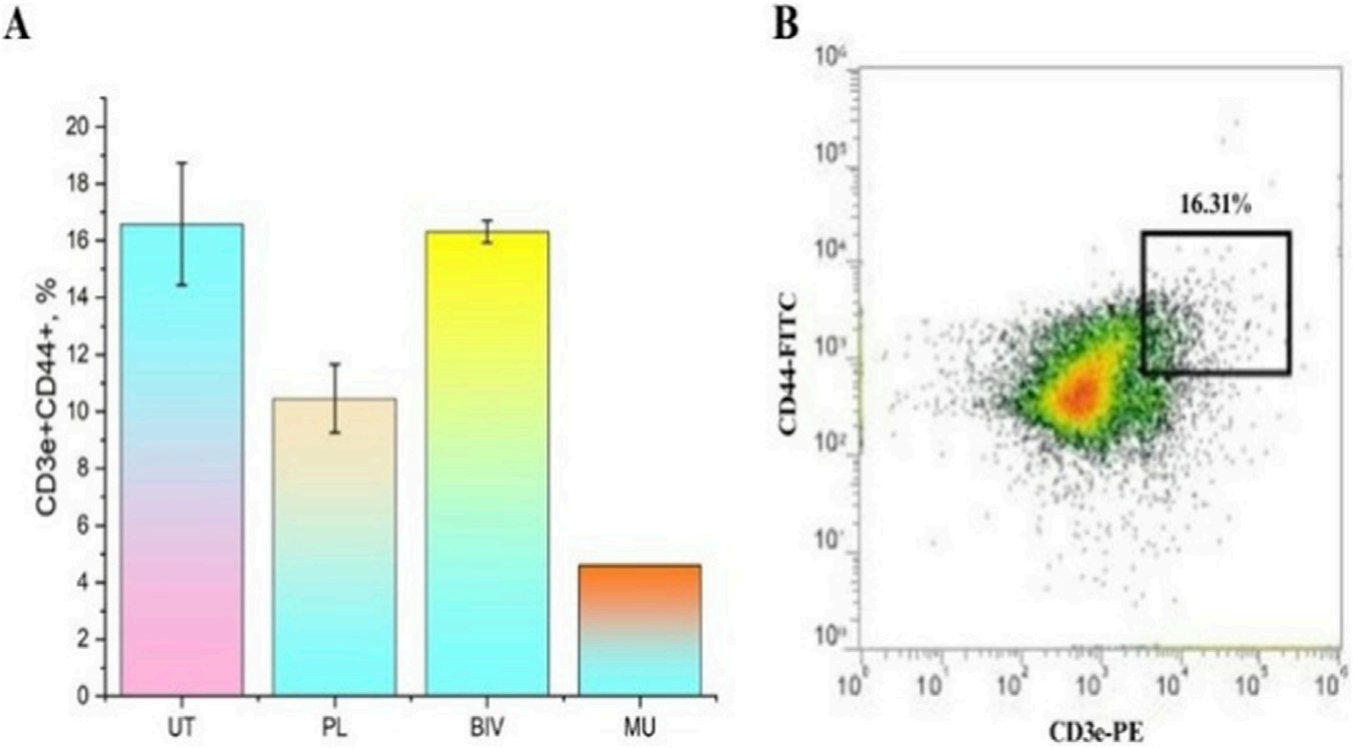
CD3ε^+^CD44^+^ recirculating T_mem_–lymphocytes levels in bone marrow. **(A)** Mean values (M ± SD) by experimental groups: UT, intact; MU, methyluracil; PL, placebo; BIV. **(B)** Representative flow cytometry dot plot illustrating CD3ε^+^CD44^+^ T_mem_ - lymphocytes in the BIV group (16.31%).

In contrast, the content of CD3ε ^+^CD44^+^ T_mem_ memory lymphocytes in the bone marrow was markedly elevated in the BIV group (Figure 1). Their level was comparable to that of the intact group and exceeded the MU group by 3.58-fold (P = 0.00005, F > F_crit_, 12865.4 > 18.51). This observation should be interpreted with caution because T_mem_ -lymphocytes represent a recirculating cell population. Therefore, the increased proportion detected in bone marrow likely reflects redistribution rather than local restoration of this population.

Pre-T-lymphocytes are known to migrate from the bone marrow to the thymus, where proliferation, positive and negative selection, and differentiation occur. Accordingly, the functional recovery of T-lymphopoiesis within the lymphomyeloid system is more accurately evaluated by thymic output and by the distribution of mature T-lymphocytes in regional lymphomyeloid organs rather than by bone marrow indices alone.

### 3.3 Effect of BIV on thymic T-lymphopoiesis

Thymic T-lymphopoiesis was evaluated under conditions of CPh-induced T-lymphopoiesis suppression and following treatment with BIV. The analysis included the total pool of CD3ε ^+^CD19^−^ thymocytes, single-positive (SP) CD3ε^+^CD4^+^ and CD3ε^+^CD8a^+^ thymocytes, activated CD4^+^CD25^+^ T helper (Th_act_) cells, and FoxP3^+^CD4^+^CD25^+^ regulatory T (T_reg_) cells.

**FIGURE 2:**
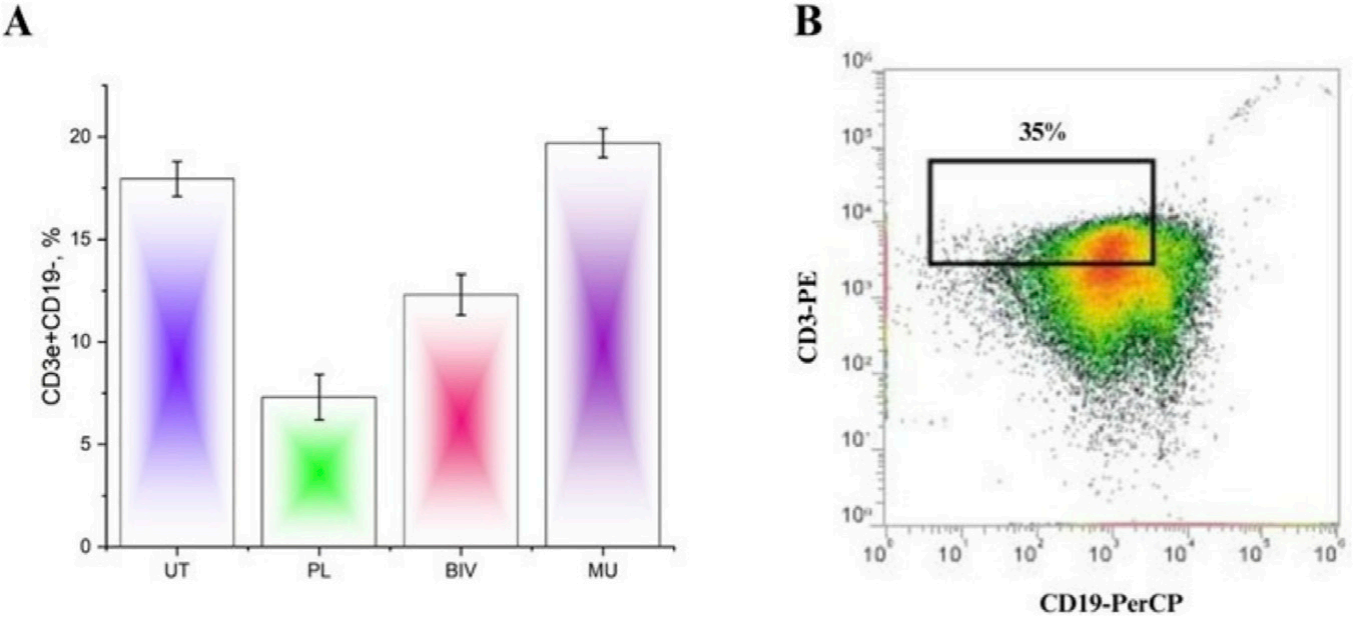
Total CD3ε^+^CD19^−^ T-lymphocyte levels in the thymus. **(A)** Mean values (M ± SD) across experimental groups (one-way ANOVA): UT, intact; MU, methyluracil; PL, placebo; BIV. **(B)** Representative flow cytometry plot of CD3ε^+^CD19^−^ thymocytes in the MU group (35%).

In intact animals, the proportion of total CD3ε ^+^CD19^−^ thymocytes was (17.95 ± 0.85) %. CPh administration resulted in a marked reduction of this population by 2.45-fold, corresponding to a 59.33% decrease relative to baseline (P = 0.00008, F > F_crit_, 12687.4 > 18.51), indicating profound thymic suppression (Figure 2).

**FIGURE 3:**
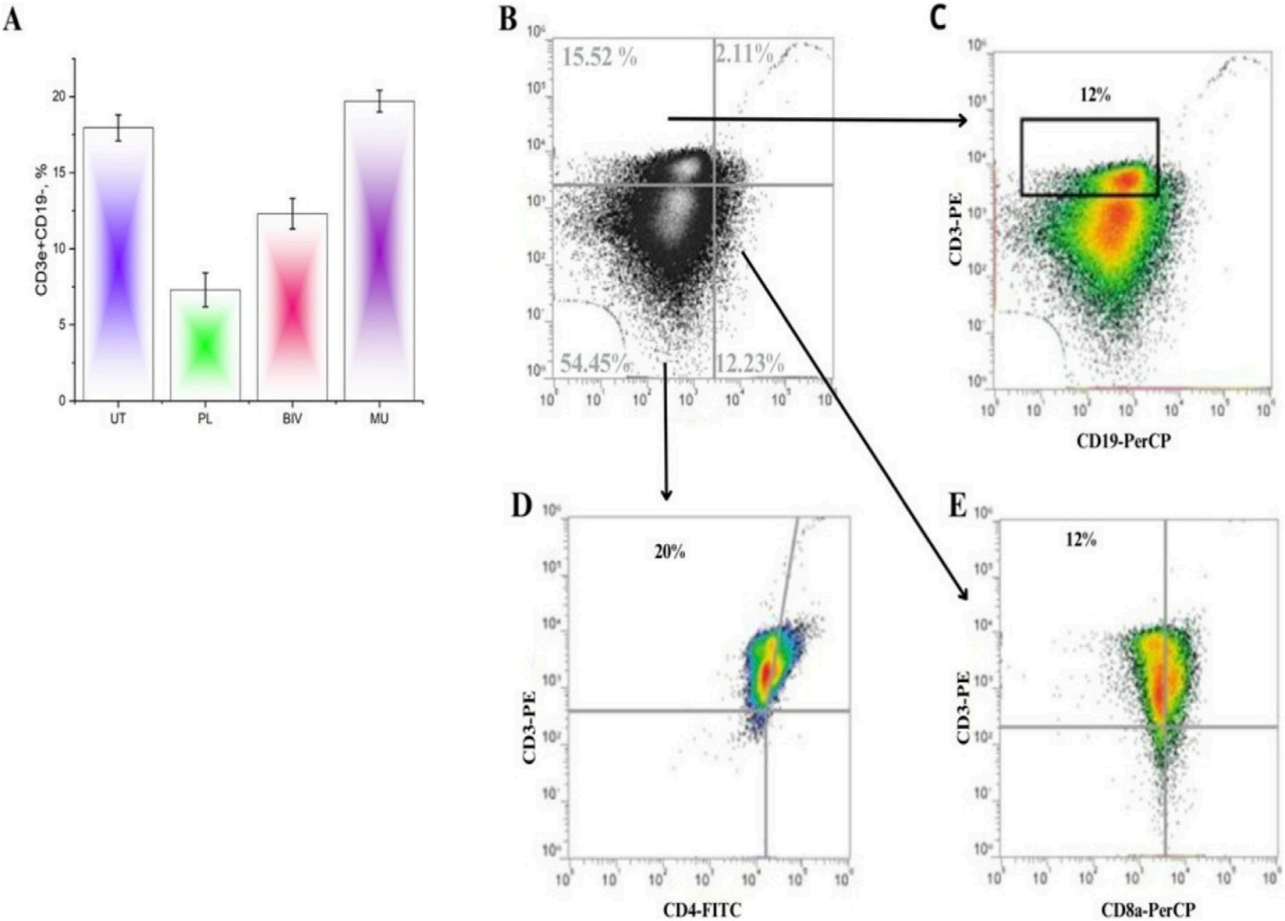
CD3ε^+^CD19^−^ thymocytes and SP CD3ε^+^CD4^+^ and SP CD3ε^+^CD8a^+^ thymocyte subpopulations levels. **(A)** Mean values (M ± SD) between groups (one-way ANOVA):UT, intact; MU, methyluracil; PL, placebo; BIV. **(B)** Representative flow cytometry plot in the BIV group showing CD3ε^+^CD19^−^ thymocytes (15.52%) and CD19^+^ cells (2.11%). **(C)** SP CD3ε^+^CD4^+^ thymocytes (12%). **(D)** SP CD3ε^+^CD8a^+^ thymocytes (20%). **(E)** SP CD3ε^+^CD8a^+^ thymocytes in the BIV group (12%).

Within the CD3ε ^+^CD19^−^ thymocyte compartment of intact animals, SP CD3ε ^+^CD4^+^ thymocytes accounted for (42.76 ± 3.01) %, while SP CD3ε^+^CD8a^+^ thymocytes constituted 28.41% ± 0.47%. CPh exerted a particularly strong suppressive effect on the CD4^+^ lineage. The proportion of SP CD3ε^+^CD4^+^ thymocytes decreased by 5.88-fold, representing an 82.99% reduction (P = 0.000008, F > F_crit_, 14892 > 18.51), and reached only (7.27 ± 0.26) %, reflecting near depletion of the thymic helper T-cell pool (Figure 3). A pronounced decline was also observed in SP CD3ε^+^CD8a^+^ thymocytes, which decreased from (28.41 ± 0.47) % to (10.20 ± 0.57) %, corresponding to a 2.78-fold reduction (64.09 ± 0.9) % (P = 0.0000005, F > F_crit_, 16652 > 18.51).

In contrast to the depletion of SP thymocytes, CPh increased the proportion of recirculating CD4^+^CD25^+^ Th_act_ cells. Their level rose from (2.34 ± 0.21) % in intact animals to (5.43 ± 0.57) %, representing a 2.32-fold increase (132.05 ± 1.4) % (P = 0.00002, F > F_crit_, 13612.5 > 18.51). At the same time, the proportion of FoxP3^+^CD4^+^CD25^+^ T_reg_ cells declined from (12.07 ± 0.67) % in intact animals to 9.76% ± 0.57% in the PL group, corresponding to a 19.13% reduction (P = 0.00003, F > F_crit_, 12546.5 > 18.51).

**FIGURE 4:**
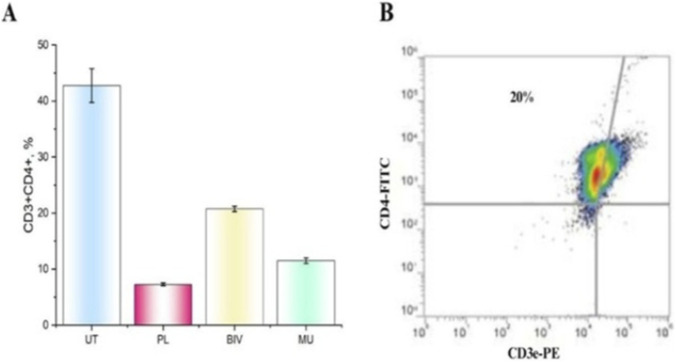
SP CD3ε^+^CD4^+^ thymocyte levels in the thymus. **(A)** Mean values (M ± SD) between groups (one-way ANOVA): UT, intact; MU, methyluracil; PL, placebo; BIV. **(B)** Representative flow cytometry plot of SP CD3ε^+^CD4^+^ thymocytes in the BIV group (20%).

BIV exhibited pronounced restorative activity toward SP thymocyte populations. The compound significantly increased the level of SP CD3ε^+^CD4^+^ thymocytes to 20.75% ± 0.48%, which was 2.85-fold higher than in the PL group (P = 0.0000001, F > F_crit_, 14622.5 > 18.51) and 1.8-fold higher than in the MU group (P = 0.0001, F > F_crit_, 182.3 > 18.51), although it remained 2.06-fold lower than the intact control (P = 0.0000007, F > F_crit_, 14866.2 > 18.51) (Figure 4).

**FIGURE 5:**
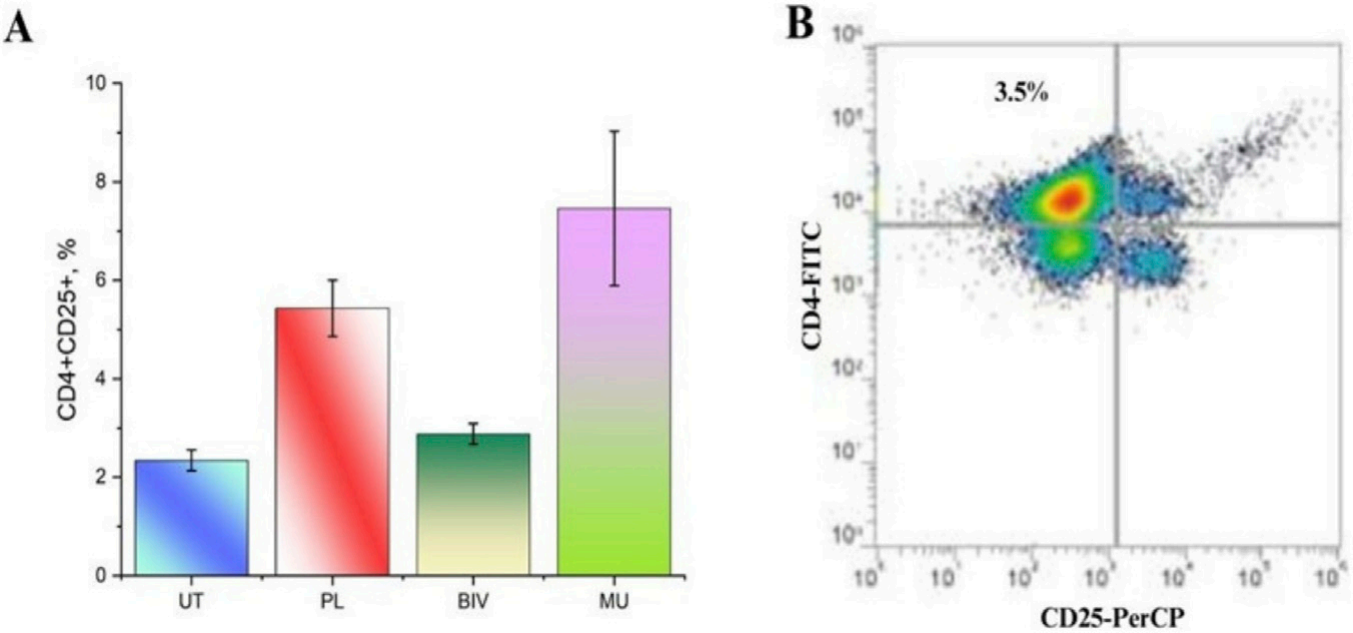
CD4^+^CD25^+^ Th_act_ -lymphocyte levels in the thymus. **(A)** Mean values (M ± SD) between groups (one-way ANOVA): UT, intact; MU, methyluracil; PL, placebo; BIV. **(B)** Representative flow cytometry plot of CD4^+^CD25^+^ Th_act_ thymocytes in the BIV group (3.5%).

Regarding SP CD3ε ^+^CD8a^+^ thymocytes, BIV demonstrated restorative activity comparable to MU. In both treatment groups, CD8a^+^ thymocyte levels increased relative to PL but did not reach intact values, remaining 2.31-fold lower than baseline (P = 0.00007, F > F_crit_, 12123.5 > 18.51). While restoration of SP CD4^+^ and SP CD8a^+^ thymocytes followed a progressive pattern, the response of CD4^+^CD25^+^ Th_act_ cells showed a different dynamic. CPh elevated Th_act_ levels from (2.34 ± 0.21) % to (5.43 ± 0.57) %. Treatment with BIV reduced this population to (2.88 ± 0.21) %, effectively restoring Th_act_ levels to the physiological range observed in intact animals. In contrast, MU caused excessive stimulation, increasing CD4^+^CD25^+^ Th_act_ cells to (7.46 ± 1.57) %, which exceeded intact values by 3.18-fold (P = 0.00007, F > F_crit_, 12102.5 > 18.51) (Figure 5).

**FIGURE 6:**
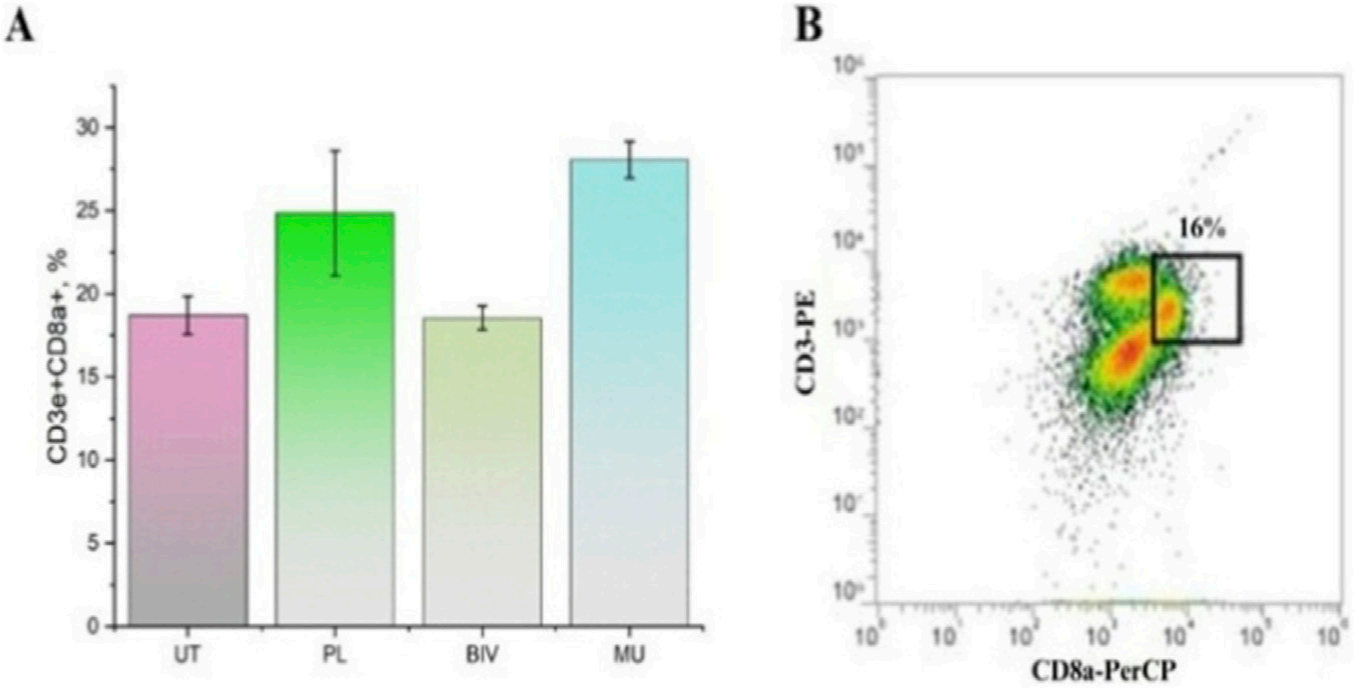
CTL-lymphocyte levels in the spleen. **(A)** Mean values (M ± SD) (one-way ANOVA) of splenic CD3ε^+^CD8a^+^ CTL-lymphocytes in experimental groups. **(B)** Representative flow cytometry plot of CD3ε^+^CD8a^+^ CTL in the BIV group. UT–untreated; PL - placebo; MU - methyluracil; BIV - BIV compound.

**FIGURE 7:**
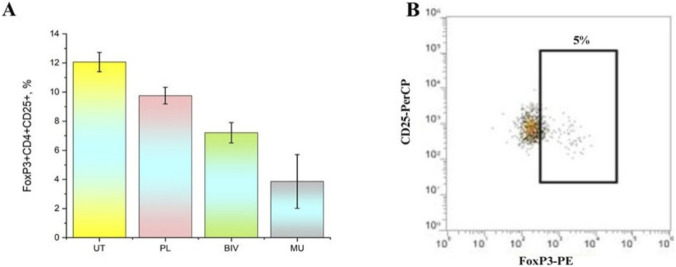
FoxP3^+^ T_reg_-lymphocyte levels in the thymus. **(A)** Mean values (M ± SD) between groups (one-way ANOVA): UT, intact; MU, methyluracil; PL, placebo; BIV. **(B)** Representative flow cytometry plot of FoxP3^+^CD4^+^CD25^+^ T_reg_ - lymphocyts in the BIV group (5%).

**FIGURE 8:**
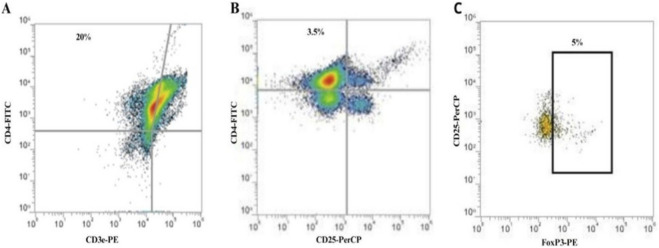
Thymic T-lymphocyte subpopulation levels. **(A)** Representative flow cytometry plot of SP CD3ε^+^CD4^+^ Th lymphocytes in the BIV group (20%). **(B)** CD4^+^CD25^+^ Th_act_-lymphocytes (3.5%). **(C)** FoxP3^+^CD4^+^CD25^+^ T_reg_ - lymphocytes (5%).

Within the CD4^+^CD25^+^ gate, CPh exposure was associated with a decrease in FoxP3^+^CD4^+^CD25^+^ T_reg_ cells, from (12.07 ± 0.67) % in intact animals to (9.76 ± 0.57) % (Figure 6). BIV induced a moderate further reduction of this population to (7.21 ± 0.70) %, corresponding to a 1.35-fold decrease (P = 0.00003 F > F_crit_, 11204.1 > 18.51). MU caused a more pronounced and potentially unfavorable effect, reducing T_reg_ levels below those observed in both UT and BIV-treated animals by 3.16-fold and 1.86-fold, respectively (P = 0.00001 F > F_crit_, 12801.5 > 18.51) (Figures 7, 8).

Overall, CPh induced severe suppression of thymic SP CD3ε^+^CD4^+^ and SP CD3ε ^+^CD8a^+^ thymocytes, accompanied by dysregulation of activated and regulatory T-cell subsets. BIV partially restored SP CD4^+^ and SP CD8a^+^ thymocyte populations, demonstrating activity comparable to MU, while exerting a more balanced immunomodulatory effect. Unlike MU, BIV normalized the elevated CD4^+^CD25^+^ Th_act_ population and avoided excessive depletion of FoxP3^+^ T_reg_ cells, suggesting a potentially more balanced immunological profile for thymic immune recovery.

### 3.4 Effect of BIV on homeostatic splenic T-lymphopoiesis

Splenic T-lymphopoiesis was evaluated by quantifying total CD3ε^+^CD19^−^ T lymphocytes and the following T-cell subpopulations: CD43^+^CD3ε^+^CD4^+^ T helper (Th) cells, CD43^+^CD3ε^+^CD8a^+^ cytotoxic T lymphocytes (CTL), activated CD4^+^CD25^+^ T helper (Th_act_) cells, CD4^+^CD25^+^FoxP3^+^ regulatory T cells (T_reg_), and CD28^+^CD44^+^CD8a^+^ memory T cells (T_mem_).

**FIGURE 9:**
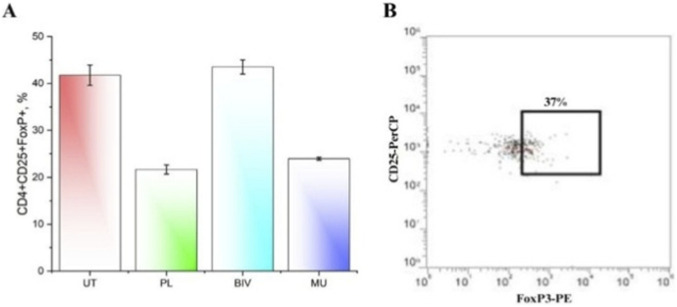
T_reg_-lymphocyte levels in the spleen. **(A)** Mean values (M ± SD) (one-way ANOVA) of CD4^+^CD25^+^FoxP3^+^ T_reg_-lymphocytes of the spleen in experimental groups. **(B)** Representative flow cytometry plot of CD4^+^CD25^+^FoxP3^+^ T_reg_ -lymphocytes in the BIV group. UT - untreated; PL - placebo; MU - methyluracil; BIV - BIV compound.

**FIGURE 10:**
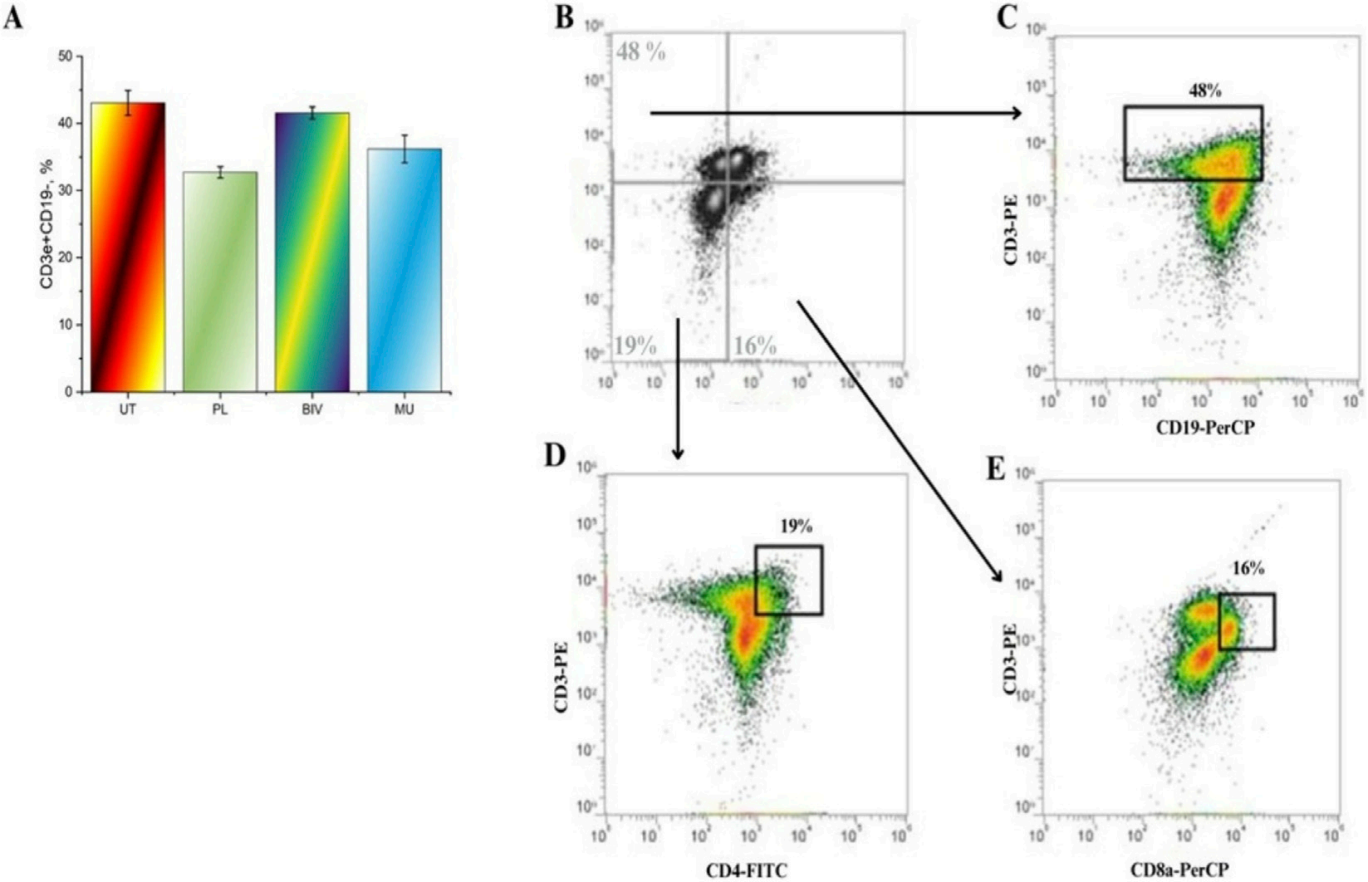
Total T-lymphocytes and T-cell subpopulations levels in the spleen. **(A)** Mean values (M ± SD) (one-way ANOVA) of splenic CD3ε^+^CD19^−^ T-lymphocytes in experimental groups. **(B)** Representative flow cytometry plot of total splenic T-lymphocytes (CD3ε^+^CD19^−^) in the BIV group. **(C)** Representative flow cytometry plot of CD3ε^+^CD4^+^ Th-lymphocytes in the BIV group. **(D)** Representative flow cytometry plot of CD3ε^+^CD8a^+^ CTL-lymphocytes in the BIV group. **(E)** Quantitative distribution of Th- and CTL-subpopulations gated from total T-lymphocytes. UT–untreated; PL–placebo; MU–methyluracil; BIV–BIV compound.

CPh exerted a less pronounced suppressive effect on splenic T-lymphocyte subpopulations compared with the thymus and bone marrow. The most prominent reduction in mitotically active lymphoid cells occurred in the bone marrow and thymus. Nonetheless, accumulating evidence indicates that the spleen remains an important site for maintaining the proliferative activity of T-cell subpopulations under cytostatic stress. CPh administration resulted in a significant reduction in total splenic CD3ε^+^CD19^−^ T lymphocytes. Their proportion decreased 1.31-fold, by 24.03%, from (43.06 ± 1.84) % in intact animals to (32.71 ± 0.84)% in the PL group (P = 0.000001; F > F_crit_, 535612.5 > 18.51) (Figure 9). Treatment with BIV markedly restored the total T-lymphocyte pool. The proportion of CD3ε^+^CD19^−^ cells increased to (41.55 ± 0.91)%, reaching values comparable to intact animals (P = 0.000001; F > F_crit_, 645248 > 18.51) and exceeding the MU group ((36.20 ± 2.05)%) by 1.46-fold (P = 0.00003; F > F_crit_, 27645.95 > 18.51) (Figure 10).

**FIGURE 11:**
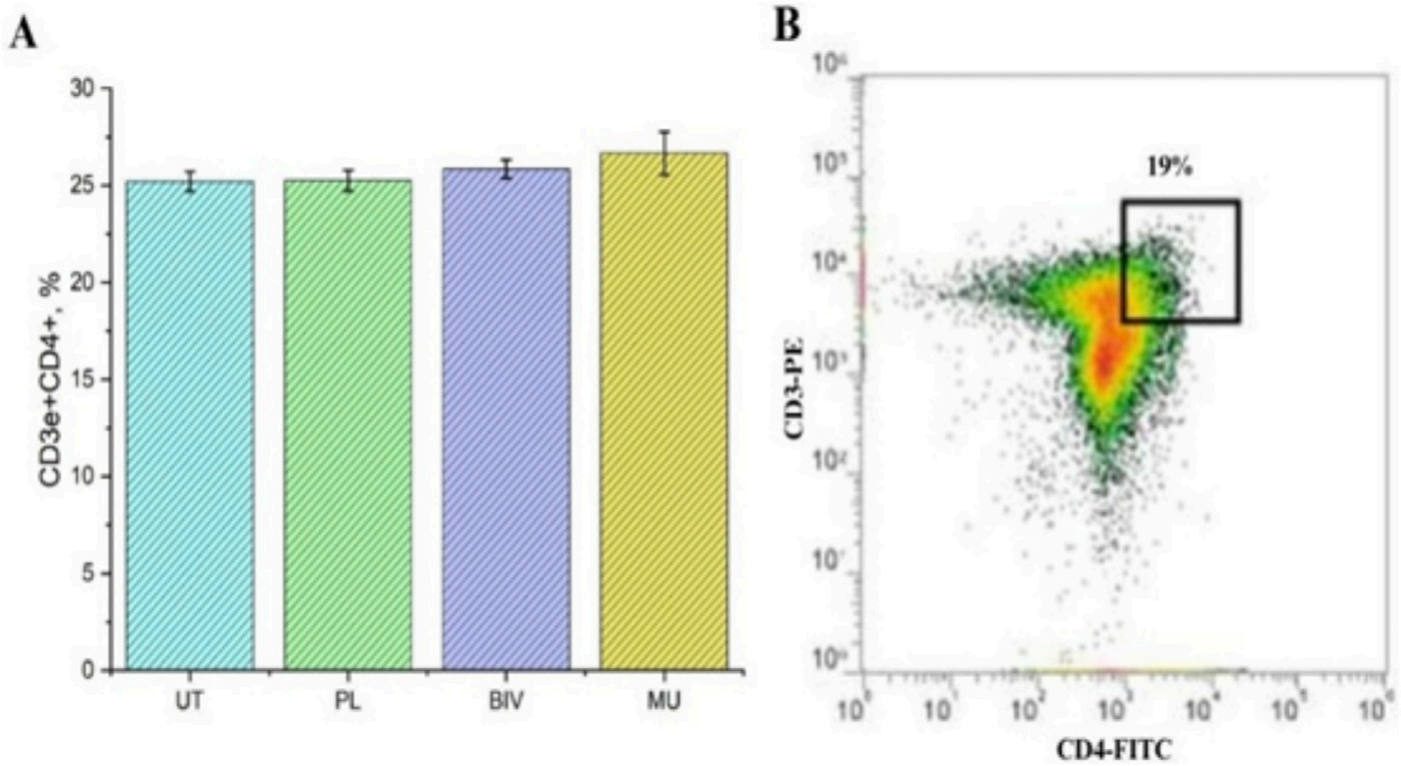
T helper (Th) lymphocyte indices in the spleen. **(A)** Mean arithmetic values (M) ± standard deviation (SD) of CD3ε+CD4+ T helper lymphocytes in the spleen across experimental groups (one-way ANOVA). **(B)** Representative flow cytometry plot showing CD3ε+CD4+ Th lymphocytes in the BIVgroup. UT ‐ intact; PL ‐ placebo; MU ‐ methyluracil; BIV ‐ BIV compound.

CPh intoxication did not significantly alter the proportion of CD43^+^CD3ε^+^CD4^+^ Th lymphocytes in the spleen. Their level remained comparable to intact controls at (25.21 ± 0.52) % (Figure 11). Similarly, BIV administration did not modify this parameter, with values of (25.85 ± 0.47) % that were indistinguishable from those observed in the PL and MU groups (26.66 ± 1.11) % and from intact animals.

In contrast, CPh caused a significant increase in the proportion of CD43^+^CD3ε^+^CD8a^+^ CTL. In the PL group, CTL levels rose from (18.71 ± 1.15) % in intact animals to (24.86 ± 3.75) %, corresponding to a 1.32-fold increase or 32.87% elevation (P = 0.000005; F > F_crit_, 189112.5 > 18.51) (Figure 6). BIV treatment did not further enhance CTL expansion but instead normalized their proportion. The level of CD43^+^CD3ε^+^CD8a^+^ CTL in the BIV group returned to values comparable with intact controls. In contrast, MU induced an excessive CTL increase, exceeding intact levels by 1.5-fold (P = 0.002; F > F_crit_, 158.1 > 18.51) (Figure 6).

**FIGURE 12:**
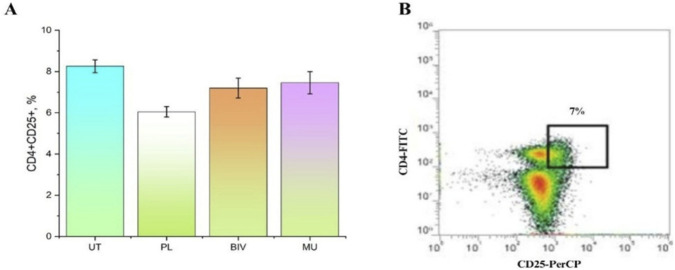
Th_act_-lymphocyte levels in the spleen. **(A)** Mean values (M ± SD) (one-way ANOVA) of CD4^+^CD25^+^ Th_act_-lymphocytes in the spleen (one-way ANOVA). **(B)** Representative flow cytometry plot of CD4^+^CD25^+^ Th_act_ -lymphocytes in the BIV group. UT - untreated; PL - placebo; MU - methyluracil; BIV - BIV compound.

CPh exposure significantly reduced the proportion of activated CD4^+^CD25^+^ Th_act cells. Their level declined from (8.26 ± 0.31)% in intact animals to (6.05 ± 0.25)%, corresponding to a 1.36-fold reduction or 26.75% decrease (P = 0.006; F > F_crit_, 142.1 > 18.51) (Figure 12).

BIV significantly promoted recovery of activated Th cells. The proportion of CD4^+^CD25^+^ Th_act_ cells increased to (7.20 ± 0.48)%, representing a 1.19-fold elevation relative to the PL group. The BIV and MU groups did not differ significantly for this parameter. Within the CD4^+^CD25^+^ gate, CPh caused a pronounced depletion of both regulatory and memory T-cell subsets. The proportion of CD4^+^CD25^+^FoxP3^+^ T_reg_ cells decreased from (41.76 ± 2.17)% in intact animals to (21.68 ± 0.98)% in the PL group, representing a 1.92-fold reduction or 48.08% decrease (P = 0.0000004; F > F_crit_, 2016032 > 18.51). In parallel, CD28^+^CD44^+^CD8a^+^ T_mem_ cells declined from (8.75 ± 1.15)% to (6.30 ± 0.77)%, corresponding to a 1.38-fold reduction or 28.0% decrease (P = 0.000003; F > F_crit_, 30012.5 > 18.51) (Figure 9).

**FIGURE 13:**
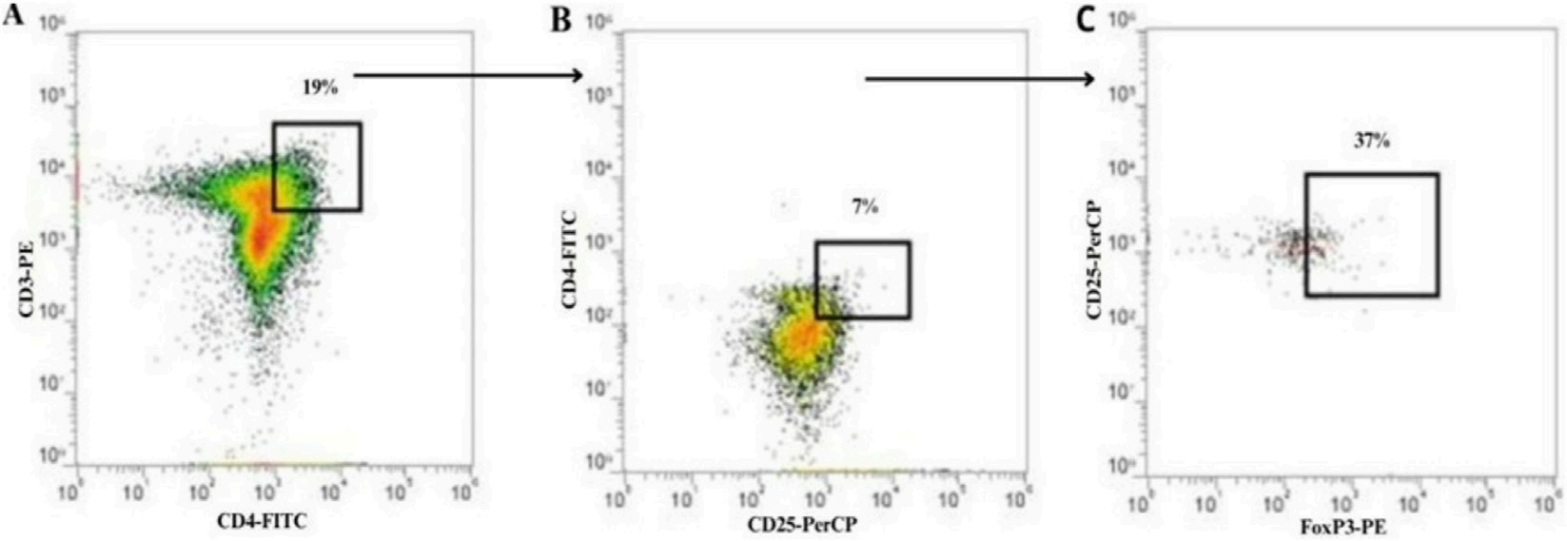
Splenic T-cell subpopulations after BIV administration. **(A)** Representative flow cytometry plot of CD3ε^+^CD4^+^ Th-lymphocytes. **(B)** Representative flow cytometry plot of CD4^+^CD25^+^ Th_act_-lymphocytes. **(C)** Representative flow cytometry plot of CD4^+^CD25^+^FoxP3^+^ T_reg_-lymphocytes.

**FIGURE 14:**
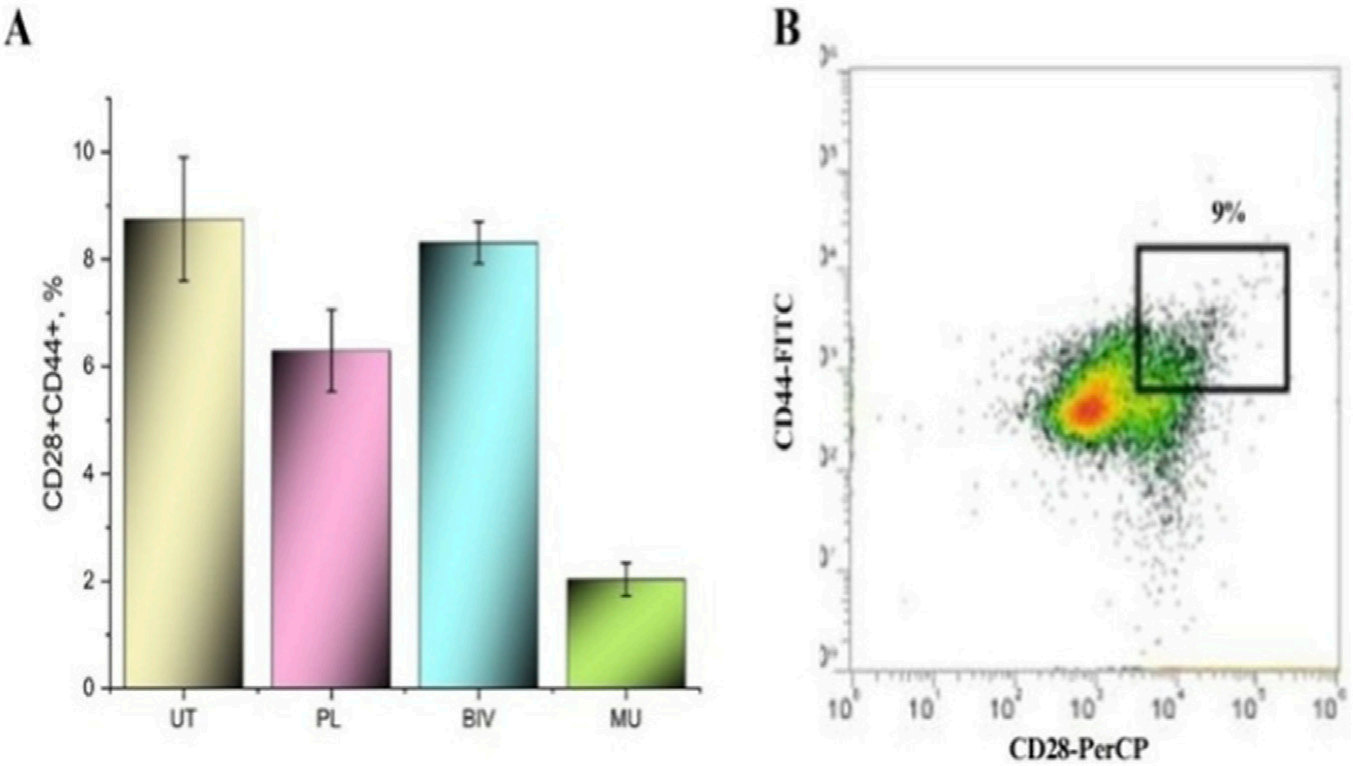
T_mem_-lymphocyte levels in the spleen. **(A)** Mean values (M ± SD) (one-way ANOVA) of CD28^+^CD44^+^CD8a^+^ T_mem_-lymphocytes in the spleen in experimental groups. **(B)** Representative flow cytometry plot of CD28^+^CD44^+^CD8a^+^ T_mem_ -lymphocytes in the BIV group. UT - untreated; PL - placebo; MU - methyluracil; BIV - BIV compound.

BIV exerted a pronounced restorative effect on regulatory T cells. The proportion of CD4^+^CD25^+^FoxP3^+^ T_reg_ cells increased to (43.52 ± 1.52)%, exceeding placebo values by 2.0-fold (100.2%) (P = 0.0000004; F > F_crit_, 2384928 > 18.51) and approaching intact control levels. MU did not significantly influence T_reg_ cell recovery (Figures 13, 14).

CPh intoxication also reduced the proportion of CD28^+^CD44^+^CD8a^+^ T_mem_ cells to (6.30 ± 0.77)% compared with (8.75 ± 1.15)% in intact animals, representing a 1.38-fold decrease (P = 0.000003; F > F_crit_, 30011.5 > 18.51) (Figure 14). BIV treatment restored the T _mem_ -cell pool to near-intact levels (8.31 ± 0.39)%. In contrast, MU further reduced the proportion of splenic CD8a^+^ T _mem_ -lymphocytes (Figure 14).

### 3.5 Effect of BIV on Bone marrow erythropoiesis

Maintenance of circulating red blood cell numbers requires continuous erythropoiesis in the bone marrow, where millions of erythrocytes are produced daily. This process is tightly regulated by intrinsic and extrinsic factors that coordinate proliferation, differentiation, and survival of erythroid precursors (Eggold and Rankin, 2019). Disruption of these regulatory mechanisms leads to pathological conditions such as anemia or polycythemia. In adult mammals, erythropoiesis occurs almost exclusively in the bone marrow under homeostatic conditions. Erythropoiesis is a highly ordered and dynamic process in which mature erythrocytes arise from hematopoietic stem and progenitor cells. Within the bone marrow, erythroid progenitors progress through distinct developmental stages, including proerythroblasts, basophilic, polychromatophilic, and orthochromatophilic erythroblasts, followed by enucleation and formation of reticulocytes. These stages are characterized by progressive reductions in cell size, increased chromatin condensation, hemoglobin accumulation, and the sequential expression of surface markers CD71 and Ter119 (Koulnis et al., 2014).

**FIGURE 15:**
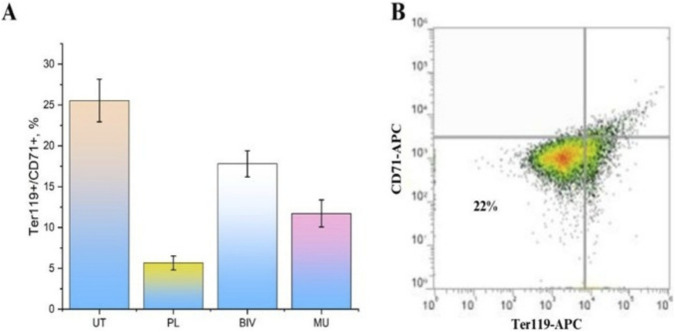
Bone marrow erythropoiesis under cyclophosphamide-induced suppression and the restorative effect of BIV. **(A)** Mean values (M ± SD) (one-way ANOVA) of Ter119^+^CD71^+^ erythroid precursor cells in the bone marrow across experimental groups. **(B)** Representative flow cytometry plot of Ter119^+^CD71^+^ erythroid cells in the BIV group. UT - untreated; PL - placebo; MU - methyluracil; BIV - BIV compound.

Among all hematopoietic lineages examined, erythroid cells were the most sensitive to CPh. CPh intoxication caused a marked depletion of erythroid precursors. In the PL group, the proportion of Ter119^+^CD71^+^ erythroid cells declined to (5.67 ± 0.85) % compared with (25.55 ± 2.60)% in intact animals (Figure 15). This corresponded to a 77.81% reduction, or a 4.51-fold decrease (P = 0.0000005; F > F_crit_, 1,976,072 > 18.51).

Clinical evidence supports a close functional relationship between erythropoiesis and bone homeostasis. Both anemic and polycythemic states are associated with an increased risk of skeletal pathology. Patients with chronic hemolytic anemias, including thalassemia, frequently exhibit reduced bone mass, fractures, and chronic bone pain (Voskaridou et al., 2018). Similarly, approximately 80% of patients with sickle cell disease present with decreased bone mineral density (Mulyana et al., 2024). Administration of BIV significantly promoted recovery of erythroid progenitors. In the BIV-treated group, the proportion of Ter119^+^CD71^+^ erythroid cells increased to (17.80 ± 1.60)%, with values ranging from 13.1% to 22.4%. This level exceeded that of the PL group ((5.67 ± 0.85)%, range (3.0–6.6)% by 3.13-fold (P = 0.000155; F > F_crit_, 6456.92 > 18.51). BIV also demonstrated greater restorative effect under the present experimental conditions compared with the reference drug MU, which yielded (11.73 ± 1.65)% Ter119^+^CD71^+^ cells (range 6.2%–17.3%), representing a 1.51-fold difference (P = 0.000623; F > F_crit_, 1603.56 > 18.51). Although BIV treatment did not fully restore erythroid cell numbers to intact control levels, it produced a substantially greater recovery than both PL and MU. This incomplete normalization is consistent with the prolonged time required for erythroid progenitor proliferation, differentiation, and maturation within the bone marrow niche (Semenza, 2023).

Overall, BIV demonstrated notable activity at the level of committed Ter119^+^CD71^+^ erythroid cells by stimulating their proliferation and survival, with efficacy exceeding that of methyluracil under conditions of cyclophosphamide-induced erythropoietic suppression.

## 4 Discussion

### 4.1 Analysis of effect of BIV on hematopoietic stem cells and T-lymphocyte populations in bone marrow

The coordinated contribution of bone marrow, thymus, and spleen to T-lymphocyte recovery following CPh-induced T-lymphopoiesis suppression remains incompletely defined. Current evidence indicates that, beyond its hematopoietic function, the bone marrow acts as an immunoregulatory niche that supports survival, recirculation, and homeostatic maintenance of mature T-cell populations. In particular, bone marrow serves as a reservoir for recirculating and memory T lymphocytes and participates in secondary immune responses rather than primary T-cell differentiation (Zhao et al., 2012).

In the present study, CPh caused marked suppression of hematopoietic and lymphoid compartments in the bone marrow. A pronounced reduction in CD117^+^ hematopoietic stem and progenitor cells was observed, confirming the high sensitivity of early proliferative pools to alkylating cytostatics. This finding is consistent with previous reports demonstrating delayed and incomplete immune reconstitution after CPh exposure, with recovery extending over several weeks and beginning at the level of bone marrow cellularity before progressing to thymic and peripheral compartments (Grinko et al., 2020).

Analysis of bone marrow T-lymphocyte composition revealed a substantial decrease in total CD3ε^+^ T-cells and CD44^+^ T_mem_-lymphocytes after CPh exposure. Bone marrow T-cell pools are known to be enriched in CD8a^+^ memory-phenotype cells that originate from the thymus and peripheral lymphoid organs. These cells undergo continuous recirculation and contribute to immune surveillance and recall responses (Zhao et al., 2012). CPh-induced lymphopenia disrupts this equilibrium and promotes compensatory homeostatic mechanisms.

Persistent T-cell deficiency is known to trigger homeostatic proliferation and conversion of naïve T lymphocytes into memory-phenotype cells in an antigen-independent manner. This process leads to accumulation of recirculating memory T cells within lymphoid niches, including bone marrow, and may contribute to immune imbalance when excessive (Sprent and Surh, 2011). These mechanisms provide a conceptual framework for interpreting the changes observed in the present study.

Evaluation of BIV activity demonstrated that the compound did not stimulate recovery of CD117^+^ hematopoietic stem cells. Importantly, the absence of mitotic activation at the level of c-Kit-expressing progenitors represents a favorable safety characteristic. Uncontrolled stimulation of this compartment is associated with leukemogenic risk, and broad-acting hematopoietic stimulants may promote pathological proliferation (Grinko et al., 2020).

BIV exerted a limited effect on the overall proportion of CD43^+^CD3ε^+^ T lymphocytes in the bone marrow and was less potent than MU in stimulating this parameter. However, within the CD3ε^+^ gate, BIV restored the proportion of CD44^+^ T_mem_-lymphocytes to levels comparable with intact animals and markedly higher than those observed after MU treatment. Given the predominantly recirculation nature of T_mem_-lymphocytes, this increase most likely reflects redistribution and retention of functional T_mem_-lymphocyte populations rather than *de novo* generation within the bone marrow (Sprent and Surh, 2011).

In contrast, MU reduced the bone marrow memory T-cell pool, shifting cell numbers toward levels associated with impaired immune competence. This divergence highlights a critical qualitative difference between the two agents. While MU promotes broad proliferative activity, BIV favors preservation of immune homeostasis by supporting physiologically relevant T-cell subsets without excessive stimulation (Grinko et al., 2020; Sprent and Surh, 2011). Recovery of T-lymphopoiesis cannot be fully assessed at the level of bone marrow alone. Pre-T lymphocytes migrate from the bone marrow to the thymus, where proliferation, selection, and differentiation determine thymic output. Consequently, functional restoration of the T-cell system is best reflected by thymic recovery and peripheral T-cell balance rather than by bone marrow indices alone (Grinko et al., 2020). In this context, the bone marrow findings support the conclusion that BIV does not act as a nonspecific proliferative agent but contributes to immune reconstitution through controlled modulation of lymphoid compartments, consistent with the thymic and splenic outcomes observed in this study.

Overall, BIV demonstrates a favorable restorative profile under CPh-induced Т-lymphopoiesis suppression. Its activity is characterized by preservation of immune balance, support of memory T-cell homeostasis, and avoidance of excessive stimulation of early hematopoietic progenitors (Zhao et al., 2012; Grinko et al., 2020; Sprent and Surh, 2011). These properties distinguish BIV from classical hematopoietic stimulants and suggest potential advantages for restoring immune competence after cytostatic injury.

### 4.2 Analysis of effect of BIV on thymic T-lymphopoiesis

Recovery of T-lymphocyte pools after CPh exposure proceeds through thymic export of newly generated T cells and peripheral homeostatic proliferation. Adult immune reconstitution during early lymphopenia phases relies mainly on peripheral expansion, since thymic output remains suppressed for an extended interval after cytotoxic conditioning (Kunstek et al., 2025). CPh produced severe thymic injury in this model. Total CD3ε^+^CD19^−^ thymocytes decreased by 59.33%, with deeper suppression of SP CD3ε^+^CD4^+^ thymocytes (82.99%) than SP CD3ε^+^CD8a^+^ thymocytes (64.09%). This pattern indicates preferential collapse of thymic helper-lineage differentiation and reduced thymic contribution to systemic T-cell recovery during the early post-treatment interval.

The thymic compartment also showed functional skewing. The proportion of recirculating CD4^+^CD25^+^ Th_act_ cells increased 2.32-fold, consistent with enrichment of activated recirculating cells during depletion of developing single-positive pools. FoxP3^+^CD4^+^CD25^+^ T_reg_ cells decreased by 19.13%, indicating impaired recovery of regulatory control during lymphopenic repopulation (Lemarquis et al., 2025). Reduced regulatory T-cell representation associates with weaker restraint of effector activation and weaker tolerance maintenance (Hall, 2016).

BIV produced a recovery profile distinct from MU in the thymus. BIV restored SP CD4^+^ thymocytes and improved SP CD8a^+^ thymocytes toward baseline trajectories. BIV also reduced Th_act_ frequency toward intact values, indicating attenuation of activation bias during thymic rebuilding. Treg depletion under BIV remained moderate relative to MU, supporting preservation of regulatory capacity during reconstitution.

MU shifted thymic recovery toward activation dominance. Th_act_ levels rose far above intact values, with pronounced depletion of FoxP3^+^ T_reg_ cells within the same gate. This effector-skewed profile aligns with reduced immune regulation and potential restriction of functional repertoire during recovery (Hall, 2016).

### 4.3 Analysis of effect of BIV on homeostatic splenic T-lymphopoiesis

Splenic findings indicate peripheral compensation during thymic failure. CPh reduced total splenic T cells and reduced activated, regulatory, and memory subsets, while cytotoxic T-cell frequency increased. Loss of splenic T_mem_ cells implies weaker formation of recall responses during early recovery, consistent with reliance on lymphopenia-driven peripheral expansion rather than stable memory maintenance (Kunstek et al., 2025).

BIV improved balance in T-lymphocytic subpopulation of the spleen. The BIV compound reduced the elevated levels of Thact lymphocytes to normal physiological values. BIV also markedly stimulated the restoration of FoxP3+ Treg lymphocyte levels, while the levels of CTL lymphocytes decreased to the physiological norm. In addition, the levels of Tmem lymphocytes in the BIV-treated group were restored to those observed in untreated (UT) animals. This pattern indicates restoration of regulation and memory compartments alongside recovery of total T-cell abundance. MU produced partial quantitative recovery of splenic total T cells, yet regulatory recovery remained limited. Persistent reduction of FoxP3^+^ T_reg_ cells coincided with higher CTL representation and reduced T_mem_ levels. This profile indicates incomplete restoration of immune regulation and adaptive memory during early post- CPh recovery.

The present study has several pharmacological and toxicological limitations. Although the acute toxicity of BIV was previously evaluated and the experimental dose was selected on the basis of the LD50 value reported by Koshetova et al. (2025), detailed pharmacokinetic parameters, absolute bioavailability, tissue distribution, and full dose-response relationships have not yet been established. In addition, systematic organ histology and serum biochemical indicators of hepatic and renal toxicity were not included in the present experimental design. Therefore, the current findings should be interpreted as evidence of preclinical hematopoietic and T-lymphopoietic restorative activity under cyclophosphamide-induced suppression, rather than as definitive proof of therapeutic safety. Further studies will include ADME profiling, pharmacokinetic/pharmacodynamic assessment, dose-response evaluation, organ histopathology, biochemical toxicity markers, and long-term safety analysis. The present findings should be interpreted within the limits of a preclinical animal study. Although BIV improved several hematopoietic and T-lymphocyte parameters after cyclophosphamide-induced suppression, these results do not establish clinical efficacy or therapeutic safety. Some observed changes, particularly in recirculating T_mem_-cell populations, may reflect redistribution and homeostatic repopulation rather than direct *de novo* lymphopoiesis. Further studies are required to define the molecular mechanism of action, pharmacokinetic and pharmacodynamic profile, long-term toxicity, dose-response relationship, and translational relevance in clinically relevant models.

### 4.4 Translational relevance across cyclophosphamide exposure regimens and study limitations

The present findings should be interpreted in the context of the different clinical patterns of cyclophosphamide exposure. Cyclophosphamide is associated with clinically important hematological toxicity, including myelosuppression, leukopenia, neutropenia, thrombocytopenia, anemia, bone marrow failure, severe immunosuppression, and increased risk of serious or fatal infections; therefore, blood-count monitoring is an essential component of clinical use (DailyMed, 2026). These toxicities provide the pharmacological background for investigating agents capable of compensating cyclophosphamide-induced hematopoietic and lymphopoietic injury.

In pulsed intravenous regimens, such as those used in autoimmune rheumatic diseases, cyclophosphamide is administered according to structured pre-administration, administration, and post-administration procedures to reduce adverse events and medication-related risks (Teles et al., 2017). In this context, a toxicity-compensating hematopoietic restorative drug would be expected to support recovery of leukopoiesis and lymphoid balance between treatment cycles without excessively stimulating early hematopoietic progenitors. The observation that BIV improved leukocyte and lymphocyte-related parameters while showing limited stimulation of CD117+ hematopoietic stem cells may therefore represent a favorable preclinical feature.

In high-dose cyclophosphamide settings, including post-transplant cyclophosphamide protocols, the therapeutic objective often involves selective immunomodulation and elimination of alloreactive T cells. Post-transplant cyclophosphamide has been described as a high-dose strategy, commonly administered at 50 mg/kg on days +3 and +4 after transplantation, followed by additional immunosuppressive agents (Bacigalupo, 2022). In such indications, a restorative adjunct would require careful timing because premature or excessive immune stimulation could theoretically interfere with the intended immunosuppressive effect. The present data do not establish whether BIV would be suitable during high-dose cyclophosphamide therapy itself, after completion of high-dose exposure, or only during the recovery phase. This remains an important unanswered question.

Continuous low-dose or metronomic cyclophosphamide has a distinct pharmacological profile. Cyclophosphamide-based metronomic chemotherapy has been reviewed as a prolonged low-dose strategy with antiangiogenic and immunomodulatory effects, especially in oncology settings (Penel et al., 2012). Under these conditions, a hematopoietic restorative agent should not simply reverse the intended immunomodulatory action of cyclophosphamide, but should ideally prevent clinically harmful cytopenia and excessive immune imbalance. The present splenic findings, including recovery of Treg and Tmem compartments and normalization of CTL and Thact levels, suggest that BIV may act as a balanced immunomodulator rather than as a nonspecific proliferative stimulant. However, this interpretation remains preliminary.

Several limitations prevent direct clinical translation. The present study used preclinical rodent models and did not compare BIV activity across continuous low-dose, pulsed, and high-dose cyclophosphamide regimens. Pharmacokinetic parameters, tissue distribution, dose-response relationships, repeated-cycle safety, and the optimal timing of BIV administration relative to cyclophosphamide exposure have not yet been established. The study also did not evaluate tumor-bearing models, autoimmune disease models, transplantation models, organ histopathology, long-term toxicity, or whether BIV affects the therapeutic efficacy of cyclophosphamide. Therefore, BIV should presently be considered a candidate hematopoietic and T-lymphopoietic restorative compound for further investigation, rather than a clinically validated detoxifying drug. Future studies should evaluate BIV in regimen-specific models of continuous, pulsed, and high-dose cyclophosphamide exposure and should determine whether hematopoietic recovery can be achieved without compromising the intended anticancer or immunosuppressive activity of cyclophosphamide.

## 5 Conclusion

In the bone marrow, cyclophosphamide (CPh) induced a pronounced reduction in the levels of c-kit cells, T lymphocytes, and recirculating Tmem cells. CPh also caused extensive thymic depletion, characterized by a marked decrease in the levels of single-positive (SP) CD3ε+CD4^+^ thymocytes (82.99%), SP CD3ε+CD8^+^ thymocytes (64.09%), and recirculating FoxP3+ Treg lymphocytes. Under conditions of CPh-induced lymphopoiesis suppression, compensatory thymic infiltration by recirculating Thact activated helper T cells (132.05%) was observed.

In the spleen, CPh induced a moderate reduction in the levels of T, Th, Thact, FoxP3+ Treg, and Tmem lymphocytes. The decrease in recirculating FoxP3+ Treg lymphocytes was associated with an increase in the level of recirculating CTL cells. A moderate reduction in Tmem lymphocytes was also observed.

The β-cyclodextrin complex of 5-benzyl-7-(o-fluorobenzylidene)-2,3-bis(o-fluorophenyl)-3,3a,4,5,6,7-hexahydro-2H-pyrazolo[4,3-c]pyridine (BIV) demonstrated T-lymphopoiesis-stimulating activity. The compound exerted limited effects on the recovery of c-kit cells and T lymphocytes in the bone marrow. In the thymus, BIV stimulated the restoration of SP CD3ε+CD4^+^ and SP CD3ε+CD8a+ thymocytes. BIV also reduced the elevated levels of Th_act_ cells to normal physiological values.

In the spleen, BIV approached the total T-lymphocyte population, as well as the Th, T_mem_, and FoxP3+ Treg lymphocyte subpopulations, physiological control levels, while reducing the levels of CTL and Thact lymphocytes to the physiological norm. Several evaluated T-lymphocyte subpopulations approached or returned to physiological control levels under the experimental conditions, although full translational validation requires further pharmacological and toxicological investigation. In addition, BIV exhibited pronounced erythropoiesis-stimulating activity.

These findings support further investigation of BIV as a potential toxicity-compensating hematopoietic and immunological restorative agent after cyclophosphamide-induced injury, particularly in studies designed to distinguish continuous low-dose, pulsed, and high-dose cyclophosphamide exposure models.

## Data Availability

The original contributions presented in the study are included in the article/Supplementary Material, further inquiries can be directed to the corresponding authors.

## Supplementary material

SUPPLEMENTARY FIGURE S1 The complex of *β*-cyclodextrin with 5-benzyl-7-(*o*-fluorobenzylidene)-2,3-bis(*o*-fluorophenyl)-3,3a,4,5,6,7-hexa-hydro-2*H*-pyrazolo[4,3-*c*]pyridine (BIV).

SUPPLEMENTARY FIGURE S2 Schematic representation of the experimental design on C57BL6/J mice.

SUPPLEMENTARY FIGURE S3 CD117^+^ hematopoietic stem cell content in bone marrow. **(A)** Mean values (M ± SD) by experimental groups: UT, intact; MU, methyluracil; PL, placebo; BIV. **(B)** Representative flow cytometry plot illustrating CD117^+^ hematopoietic stem cells in the BIV group (5%).

SUPPLEMENTARY FIGURE S4 CD3ε^+^CD43^+^ T-lymphocyte content in bone marrow. **(A)** Mean values (M ± SD) by experimental groups: UT, intact; MU, methyluracil; PL, placebo; BIV. **(B)** Representative flow cytometry histogram showing CD3ε^+^CD43^+^ T-lymphocytes in the BIV group (15%). **(C)** Representative flow cytometry dot plot showing CD3ε^+^CD43^+^ T-lymphocytes in the BIV group (20%).

## References

[B1] Abdoon A. S. S. Al-Atrash A. M. E. Soliman S. S. El-Sanea A. M. Gamal El Din A. A. Fahmy H. M. (2023). Lyophilized equine platelet-rich plasma (L-GF^equina^) antagonize the reproductive toxicity and oxidative stress induced by cyclophosphamide in female rats. J. Ovarian Res. 16 (1), 84. 10.1186/s13048-023-01161-x 37118826 PMC10141944

[B2] Abireh I. E. Ozor C. C. Mba C. E. Finbarrs-Bello E. Atuadu V. O. Aliopia E. F. (2020). Antidote to cyclophosphamide-induced myelosuppression: the Vitex doniana leaf effect. IOSR J. Dent. Med. Sci. 19, 47–50. 10.13140/RG.2.2.12285.51688

[B3] Angley M. T. Sansom L. N. Stupans I. (1995). Cyclophosphamide administered repeatedly to the Male rat and as a single dose to the female rat. Its effects on hepatic and pulmonary P450 and associated enzymes. Xenobiotica 25 (10), 1051–1062. 10.3109/00498259509061905 8578761

[B4] Aoki N. Xing Z. (2004). Use of cytokines in infection. Expert Opin. Emerg. Drugs 9 (2), 223–236. 10.1517/14728214.9.2.223 15571481

[B5] Azuma I. (1992). Review: inducer of cytokines *in vivo:* overview of field and romurtide experience. Int. J. Immunopharmacol. 14 (3), 487–496. 10.1016/0192-0561(92)90180-S 1618600

[B6] Bacigalupo A. (2022). Post-transplant cyclophosphamide: overcoming the HLA barrier to hematopoietic stem cell transplants. Haematologica 107, 1230–1231. 10.3324/haematol.2022.281256 35642483 PMC9152978

[B7] Baktybayeva L. Daulet G. Zazybin A. Yu V. Ostapchuk Y. Perfilyeva Y. (2023). Stimulation of B-lymphopoiesis by administration of a trimecaine-based ionic compound in cyclophosphamide-induced hematopoietic-depressive model. Molecules 28, 1378. 10.3390/molecules28031378 36771044 PMC9920924

[B8] Bondarchuk A. O. (2016). Morphological foundation of methyluracyl application for correction of cyclophosphamide toxic effect on rats small intestine tissues. Eksperimental’naia I Klin. Gastroenterol. 12, 83–87. 29889428

[B9] DailyMed (2026). Cyclophosphamide for Injection, USP: Full Prescribing Information. Bethesda, MD: U.S. National Library of Medicine.

[B10] Di Carlo S. Häcker G. Gentle I. E. (2022). GM-CSF suppresses antioxidant signaling and drives IL-1β secretion through NRF2 downregulation. EMBO Rep. 23 (8), e54226. 10.15252/embr.202154226 35695080 PMC9346485

[B11] Dressel H. (1977). Remarks on some properties and effects of sodium nucleinate. Dev. Biol. Stand 34, 85–92. 838153

[B12] Eggold J. T. Rankin E. B. (2019). Erythropoiesis, EPO, macrophages, and bone. Bone 119, 36–41. 10.1016/j.bone.2018.03.014 29551752 PMC6139082

[B13] Eryanti Y. Hendra R. Herlina T. Zamri A. Supratman U. (2015). Synthesis of curcumin analogue, *n*-H and *n*-benzil-4-piperidone and their cytotoxic activity. Procedia Chem. 17, 224–229. 10.1016/j.proche.2015.12.136

[B14] Finkel M. S. Oddis C. V. Jacob T. D. Watkins S. C. Hattler B. G. Simmons R. L. (1992). Negative inotropic effects of cytokines on the heart mediated by nitric oxide. Science 257, 387–389. 10.1126/science.1631560 1631560

[B15] Giemsa G. (1904). Eine Vereinfachung und Vervollkommnung meiner Methylenazur-Methylenblau-Eosin-Färbemethode zur Erzielung der Romanowsky-Nochtschen Chromatinfärbung. Cent. Bakt. 37, 308–311.

[B16] Gimadieva A. R. Myshkin V. A. Mustafin A. G. Chernyschenko Y. N. Fattakhov A. K. Abdrakhmanov I. B. (2013). 5-amino-6-methyluracil is a promising pyrimidine antioxidant. Dokl. Biol. Sci. 448, 7–9. 10.1134/S0012496613010110 23479008

[B17] Grafeneder J. Derhaschnig U. Eskandary F. Buchtele N. Sus N. Frank J. (2022). Micellar curcumin: pharmacokinetics and effects on inflammation markers and PCSK-9 concentrations in healthy subjects in a double-blind, randomized, active-controlled, crossover trial. Mol. Nutr. Food Res. 66, e2200139. 10.1002/mnfr.202200139 36101515 PMC9787856

[B18] Grinko E. K. Donetskova A. D. Mukhina E. A. Andreeva O. S. Sharova N. I. Komogorova V. V. (2020). Dynamics of recovery of T-lymphocytes after induction of lymphopenia with cyclophosphane. Immunology 41, 282–292. 10.33029/0206-4952-2020-41-4-285-294

[B19] Guevara N. A. Flores Chang M. M. Castelar J. Sequeira H. Berger J. (2023). A challenging diagnosis of febrile pancytopenia in a patient with a history of autoimmune disease. Cureus 15 (3), e35956. 10.7759/cureus.35956 37038578 PMC10082673

[B20] Hall B. M. (2016). CD4+CD25+ T regulatory cells in transplantation tolerance: 25 years on. Transplantation 100, 2533–2547. 10.1097/TP.0000000000001436 27861285

[B21] Hanagata N. (2012). Structure-dependent immunostimulatory effect of CpG oligodeoxynucleotides and their delivery system. Int. J. Nanomedicine 7, 2181–2195. 10.2147/IJN.S30197 22619554 PMC3356174

[B22] Haselwanter P. Bal C. Gompelmann D. Idzko M. Prosch H. Zauner C. (2023). Sustained treatment response after intravenous cyclophosphamide in a patient with therapy-resistant COVID-19 acute respiratory distress syndrome: a case report. J. Clin. Med. 12 (17), 5506. 10.3390/jcm12175506 37685571 PMC10488024

[B23] He X. Y. Yan L. Xu X. P. Huang S. Yan D. L. Qin X. M. (2018). Investigation on positive effect and possible mechanisms of lvjiao buxue granules treatment on cyclophosphamide-induced leukopenia mice through 1H-NMR based metabolomics approach. Chin. Tradit. Herb. Drugs 49, 2282–2290. 10.7501/j.issn.0253-2670.2018.10.008

[B24] Huyan X. H. Lin Y. P. Gao T. Chen R. Y. Fan Y. M. (2011). Immunosuppressive effect of cyclophosphamide on white blood cells and lymphocyte subpopulations from peripheral blood of Balb/c mice. Int. Immunopharmacol. 11, 1293–1297. 10.1016/j.intimp.2011.04.011 21530682

[B25] Kabdualiev A. K. Ivanov R. A. Syzdykov Zh. S. Umirbekov K. B. Makhmutov M. B. (2007). Correction of immunological disorders with mexidol during operational stress. Allergology Immunology 8, 166–168.

[B26] Kato K. Takeuchi A. Akashi K. Eto M. (2020). Cyclophosphamide-induced tolerance in allogeneic transplantation: from basic studies to clinical application. Front. Immunol. 10, 3138. 10.3389/fimmu.2019.03138 32082305 PMC7005582

[B27] Klinman D. M. Yi A. K. Beaucage S. L. Conover J. Krieg A. M. (1996). CpG motifs present in bacteria DNA rapidly induce lymphocytes to secrete interleukin 6, interleukin 12, and interferon gamma. Proc. Nat. Acad. Sci. U. S. A. 93, 2879–2883. 10.1073/pnas.93.7.2879 8610135 PMC39727

[B28] Koshetova Z. Daulet G. Ten A. Koizhaiganova R. Baktybayeva L. Zharkynbek T. (2025). Design, characterization, and hematopoietic efficacy of a fluorinated pyrazolopiperidine inclusion complex. Molecules 30, 4047. 10.3390/molecules30204047 41157064 PMC12566042

[B29] Koulnis M. Porpiglia E. Hidalgo D. Socolovsky M. (2014). “Erythropoiesis: from molecular pathways to system properties,” *in* A Systems Biology Approach to Blood. Advances in Experimental Medicine and Biology. Editors Corey S. Kimmel M. Leonard J. (New York: Springer), 844, 37–58. 10.1007/978-1-4939-2095-2_3 25480636

[B30] Kunstek H. Kieviet J. Lindemans C. de Koning C. Nierkens S. (2025). Thymic peptides in immune reconstitution and clinical outcome after allogeneic hematopoietic cell transplantation. Blood Neoplasia 2, 100090. 10.1016/j.bneo.2025.100090 40949771 PMC12423692

[B31] Lee K. M. C. Achuthan A. A. Hamilton J. A. (2020). GM-CSF: a promising target in inflammation and autoimmunity. Immunotargets Ther. 9, 225–240. 10.2147/ITT.S262566 33150139 PMC7605919

[B32] Lemarquis A. L. Kousa A. I. Argyropoulos K. V. Jahn L. Gipson B. Pierce J. (2025). Recirculating regulatory T cells mediate thymic regeneration through amphiregulin following damage. Immunity 58, 397–411.e6. 10.1016/j.immuni.2025.01.006 39892391 PMC11932356

[B33] Liu B. Tan W. Barsoum A. Gu X. Chen K. Huang W. (2010). IL-17 is a potent synergistic factor with GM-CSF in mice in stimulating myelopoiesis, dendritic cell expansion, proliferation, and functional enhancement. Exp. Hematol. 38 (10), 877–884. 10.1016/j.exphem.2010.06.004 20600582

[B34] Liu B. Luo Y. Luo D. Zhou W. Zhang Y. He R. (2017). Treatment effect of low intensity pulsed ultrasound on leukopenia induced by cyclophosphamide in rabbits. Am. J. Transl. Res. 9, 3315–3325. 28804549 PMC5553881

[B35] Liu Y. Pan Y. Hu Z. Wu M. Wang C. Feng Z. (2020). Thymosin alpha 1 reduces the mortality of severe COVID-19 by restoration of lymphocytopenia and reversion of exhausted T cells. Clin. Infect. Dis. 71, 2150–2157. 10.1093/cid/ciaa630 32442287 PMC7314217

[B36] Lundqvist A. Smith A. L. Takahashi Y. Wong S. Bahceci E. Cook L. (2013). Differences in the phenotype, cytokine gene expression profiles, and *in vivo* alloreactivity of T cells mobilized with plerixa for compared with G-CSF. J. Immunol. 191, 6241–6249. 10.4049/jimmunol.1301148 24244025 PMC3892143

[B37] Luo W. Liu Y. Qin H. Zhao Z. Wang S. He W. (2024). Nitrogen-containing heterocyclic drug products approved by the FDA in 2023: synthesis and biological activity. Eur. J. Med. Chem. 279, 116838. 10.1016/j.ejmech.2024.116838 39255645

[B38] Makheja K. D. Maheshwari B. K. Arain S. Kumar S. Kumari S. Vikash. (2013). The common causes leading to pancytopenia in patients presenting to tertiary care hospital. Pak. J. Med. Sci. 29 (5), 1108–1111. 10.12669/pjms.295.3458 24353701 PMC3858928

[B39] Meade M. G. Bolaños-Meade J. (2024). The history of haploidentical stem cell transplantation: a trip from the bench to the bedside. Hematology 29, 2346401. 10.1080/16078454.2024.2346401 38687632 PMC11285319

[B40] Mulyana A. M. Rakhmawati W. Pramukti I. Lukman M. Wartakusumah R. Mediani H. S. (2024). Bone disease among children with sickle cell disease: a scoping review of incidence and interventions. J. Multidiscip. Healthc. 17, 3235–3246. 10.2147/JMDH.S475371 39006879 PMC11246032

[B41] Nagai K. (2023). Immunosuppressive agent options for primary nephrotic syndrome: a review of network meta-analyses and cost-effectiveness analysis. Med. Kaunas. 59, 601. 10.3390/medicina59030601 36984602 PMC10054564

[B42] Order of the Minister of Healthcare of the Republic of Kazakhstan (2020). On approval of the rules for the assessment of materials and compliance of preclinical (non-clinical) studies with good Laboratory Practice (GLP) requirements of the Republic of Kazakhstan And/or the Eurasian economic union within the framework of pharmaceutical inspection. Available online at: https://adilet.zan.kz/eng/docs/V2000021596 (Accessed January 22, 2026).

[B43] Papalois A. E. Barbatis C. Chrysikos D. Korontzi M. Sideris M. Pittaras T. (2019). Treatment with Molgramostim (recombinant human granulocyte-macrophage colony stimulating factor, rhugm-csf, Mielogen) and lenograstim (granulocyte-colony stimulating factor) improves experimental colitis in rats. Biomed. Res. Int. 2019, 8298192. 10.1155/2019/8298192 31687401 PMC6803744

[B44] Penel N. Adenis A. Bocci G. (2012). Cyclophosphamide-based metronomic chemotherapy: after 10 years of experience, where do we stand and where are we going? Crit. Rev. Oncology/Hematology 82 (1), 40–50. 10.1016/j.critrevonc.2011.04.009 21641231

[B45] Purygin P. P. Kuz’mina V. E. Vishnyakov V. V. (2004). Synthesis of cyano derivatives of pyrimidine bases and their effect on blood leukocyte system. Pharm. Chem. J. 38, 662–664. 10.1007/s11094-005-0055-6

[B46] Qu X. Zhang Q. Zhang C. Sun J. Du S. Liang C. (2024). Effect of chondroitin sulfate modified polyethyleneimine on mediating oligodeoxynucleotide YW002 in the treatment of periodontitis. RSC Adv. 14 (28), 20328–20338. 10.1039/d4ra00884g 38919285 PMC11197841

[B47] Quan X. Chen H. Liang S. Yang C. Yao C. Xu Y. (2022). Revisited cyclophosphamide in the treatment of lupus nephritis. Biomed. Res. Int. 2022, 8345737. 10.1155/2022/8345737 35707391 PMC9192236

[B48] Robinson T. M. O’Donnell P. V. Fuchs E. J. Luznik L. (2016). Haploidentical bone marrow and stem cell transplantation: experience with post-transplantation cyclophosphamide. Semin. Hematol. 53 (2), 90–97. 10.1053/j.seminhematol.2016.01.005 27000732 PMC4806368

[B49] Sato-Okabayashi Y. Isoda K. Heissig B. Kadoguchi T. Akita K. Kitamura K. (2020). Low-dose oral cyclophosphamide therapy reduces atherosclerosis progression by decreasing inflammatory cells in a murine model of atherosclerosis. Int. J. Cardiol. Heart Vasc. 10 (28), 100529. 10.1016/j.ijcha.2020.100529 32577494 PMC7300380

[B50] Semenza G. L. (2023). Regulation of erythropoiesis by the hypoxia-inducible factor pathway: effects of genetic and pharmacological perturbations. Annu. Rev. Med. 74, 307–319. 10.1146/annurev-med-042921-102602 35773226

[B51] Sharma P. Georgy J. T. Andrews A. G. John A. O. Joel A. Chacko R. T. (2022). Anemia requiring transfusion in breast cancer patients on dose-dense chemotherapy: prevalence, risk factors, cost and effect on disease outcome. Support. Care Cancer 30, 5519–5526. 10.1007/s00520-022-06970-2 35314996 PMC8937495

[B52] Singh M. Singh V. Singh D. P. Bohra G. K. Misra A. K. (2021). Hemorrhagic manifestation in different etiologies of pancytopenia: a prospective, cross-sectional study. J. Fam. Med. Prim. Care 10 (2), 804–808. 10.4103/jfmpc.jfmpc_1117_20 34041080 PMC8138370

[B53] Sprent J. Surh C. D. (2011). Normal T cell homeostasis: the conversion of naive cells into memory-phenotype cells. Nat. Immunol. 12, 478–484. 10.1038/ni.2018 21739670 PMC3434123

[B54] Steinbrecht S. Kiebist J. König R. Thiessen M. Schmidtke K. U. Kammerer S. (2020). Synthesis of cyclophosphamide metabolites by a peroxygenase from *marasmius Rotula* for toxicological studies on human cancer cells. Amb. Express 10, 128. 10.1186/s13568-020-01064-w 32683510 PMC7368878

[B55] Teles K. A. Medeiros-Souza P. Lima F. A. C. Araújo B. G. D. Lima R. A. C. (2017). Cyclophosphamide administration routine in autoimmune rheumatic diseases: a review. Rev. Bras. Reumatol. 57, 596–604. 10.1016/j.rbre.2016.09.008 29173694

[B56] Thwaites R. S. Sanchez Sevilla Uruchurtu A. Siggins M. K. Liew F. Russell C. D. Moore S. C. (2021). Inflammatory profiles across the spectrum of disease reveal a distinct role for GM-CSF in severe COVID-19. Sci. Immunol. 6, eabg9873. 10.1126/sciimmunol.abg9873 33692097 PMC8128298

[B57] Tian J. Zhao H. Gao Y. Wang Q. Xiang H. Xu X. (2021). Branched-chain amino acids catabolism pathway regulation plays a critical role in the improvement of leukopenia induced by cyclophosphamide in 4T1 tumor-bearing mice treated with lvjiaobuxue granule. Front. Pharmacol. 12, 657047. 10.3389/fphar.2021.657047 34759816 PMC8573099

[B58] Tolkacheva V. V. Kazakhmedov E. R. Kobalava Zh. D. Galochkin S. A. Shchulkin A. V. (2021). Effect of mexidol on the quality of life and functional status of patients with chronic cerebral ischemia and chronic heart failure with reduced left ventricular ejection fraction. Kardiologiya I Serdechno-Sosudistaya Khirurgiya 14, 80. 10.17116/kardio20211401180

[B59] Voskaridou E. Dimopoulou M. Terpos E. (2018). Osteoporosis in thalassaemia. Thalass. Rep. 8, 7487. 10.4081/thal.2018.7487

[B60] Yang S. Jin H. Zhao Z. (2019). The protective effect of cantharidinate sodium on cyclophosphamide-induced leukopenia in mice. Int. J. Clin. Exp. Med. 12 (6), 7311–7316.

[B61] Yu V. Ten A. Baktybayeva L. Rakhatov D. Zharkynbek T. Tsay K. (2018). Synthesis and biological evaluation of 1,3,8-triazaspiro[4.5]decane-2,4-dione derivatives as myelostimulators. J. Chem. 2018, 7346835–7346839. 10.1155/2018/7346835

[B62] Zhao E. Xu H. Wang L. Kryczek I. Wu K. Hu Y. (2012). Bone marrow and the control of immunity. Cell. Mol. Immunol. 9, 11–19. 10.1038/cmi.2011.47 22020068 PMC3251706

[B63] Zhao Y. Kilian C. Turner J. E. Bosurgi L. Roedl K. Bartsch P. (2021). Clonal expansion and activation of tissue-resident memory-like Th17 cells expressing GM-CSF in the lungs of severe COVID-19 patients. Sci. Immunol. 6, eabf6692. 10.1126/sciimmunol.abf6692 33622974 PMC8128299

[B64] Zhu L. Dou Y. Bjorner M. Ladiges W. (2020). Development of a cyclophosphamide stress test to predict resilience to aging in mice. Geroscience 42 (6), 1675–1683. 10.1007/s11357-020-00222-z 32613492 PMC7732896

